# Supplementary material for: Evaluating the effectiveness of a brief digital procrastination intervention targeting university students in Sweden: study protocol for the Focus randomised controlled trial
Source: BMJ Open. 2023 Jul 21;13(7):e072506. doi: 10.1136/bmjopen-2023-072506 (PMC10364182; doi:10.1136/bmjopen-2023-072506)
Supplement: Supplementary data [file bmjopen-2023-072506supp001.pdf]

APPENDIX A – INFORMED CONSENT

We would like to ask you to participate in a research project. On this page you can find information about the project and what participation entails.

WHY ARE WE DOING THIS STUDY AND WHY DO YOU WANT ME TO PARTICIPATE?

Focus is a research project which aims to estimate the effects of a brief digital procrastination intervention on procrastination, anxiety and stress symptoms, and lifestyle behaviours among university students in Sweden. The intervention is delivered online and since you are a college or university student enrolled at a university in Sweden, we would like to ask you if you would like to participate in the study.

The research project is conducted at Linköping University in Sweden, please find contact details for the primary investigator below.

WHAT IS EXPECTED OF ME?

Those who consent to take part in the study will first be asked to complete a short, online questionnaire about their current levels of procrastination. Participants will thereafter receive feedback on their current level of procrastination. Depending on which version of Focus you have been assigned to, you might also receive access to resources designed to support you change your procrastination behaviour. After two months, all participants will, again, be asked to complete a follow-up questionnaire regarding their current levels of anxiety and stress, procrastination, and lifestyle behaviours (via e-mail). The questionnaires take 5-10 minutes to complete.

WILL I BE TAKING ANY RISKS BY PARTICIPATING IN THIS STUDY?

If you decide to take part in the study then you should be aware that while Focus has been designed based on current scientific evidence regarding how to support individuals to change their procrastination behaviours, however, not everyone who uses Focus will succeed in procrastinating less. This may feel like a failure and may be de-motivating. Participants should also be aware that changing behaviours and challenging oneself to act differently, may initially result in discomfort and increased stress. These discomforts are passing, and in the long run the benefits of a more sustainable study situation will outweigh the discomforts.

You can always contact your student- or primary-healthcare centre if you feel any discomforts, want more help, or wish to consult a professional regarding your mental or physical health. You can also contact 1177 if you have any questions about your health.

WILL I BE LEAVING ANY PERSONAL INFORMATION?

The project will collect and register information about you. No unauthorized persons will have access to your answers.

Your responses to the questionnaires during the study period will be stored in a database at Linköping University. We will use an encrypted version of your e-mail address in order to connect your responses from the different questionnaires. The secret key used to decrypt your e-mail address will only be accessible by the primary investigator Marcus Bendtsen (see contact information below). Neither during analysis nor publication of findings will any information be connected to you individually.

When the project is complete, all e-mail addresses will be deleted. The information will be stored at Linköping University for 10 years. The data collection is for scientific research and is therefore motivated by public interest (GDPR EU 2016/679, Prop. 2017/18:298).

Linköping University is responsible for your data. According to EU:s data protection regulation you have the right to, free of charge, be given access to the data collected about you in the project, and have errors corrected. You may also request that your data be deleted or use of your data be restricted. However, the right to erasure and to limit the processing of personal data does not apply when the data is necessary for the current research. If you wish to be given access to your data, you should contact the primary investigator Marcus Bendtsen (see contact information below). The data protection officer can be reached at dataskyddsombud@liu.se. If you are dissatisfied with the way your personal data has been handled, you can file a complaint at the Swedish Authority for Privacy Protection.

HOW CAN I GET MORE INFORMATION ABOUT THE RESULTS FROM THIS STUDY?

After the study has been completed, findings will be published in scientific peer-reviewed journals. Findings at the individual level will not be traceable from these publications. We will not contact you after study completion, but you are welcome to contact us if you want information on findings or copies of published reports.

INSURANCE

As a participant of a research project at Linköping University you are included in the insurance the university has at Kammarkollegiet.

PARTICIPATION IS FREE

Participation is free and you can at any time decide to end it. If you decide to end your participation you will not be asked why, and it will not affect your studies. If you wish to end your participation you should contact the primary investigator Marcus Bendtsen (see contact information below).

PRIMARY INVESTIGATOR

Marcus Bendtsen, PhD
Docent in Medical Informatics
Senior Lecturer in Experimental Social Medicine and Public Health
Department of Health Medicine and Caring Sciences, Linköping University
marcus.bendtsen@liu.se, 013-286975
