# Supplementary material for: Evaluating the effectiveness of a brief digital procrastination intervention targeting university students in Sweden: study protocol for the Focus randomised controlled trial
Source: BMJ Open. 2023 Jul 21;13(7):e072506. doi: 10.1136/bmjopen-2023-072506 (PMC10364182; doi:10.1136/bmjopen-2023-072506)
Supplement: Supplementary data [file bmjopen-2023-072506supp002.pdf]

APPENDIX B – QUESTIONNAIRES

BASELINE

1. Sex:
   a. Female
   b. Male
   c. Nonbinary
   d. Other

2. Age (numerical measure)

PROCRASTINATION

3. I delay making decisions until it´s too late
   1) Very seldom or not true of me
   2) Rarely true of me
   3) Sometimes true of me
   4) Often true of me
   5) Very often true or true of me

4. Even after I make a decision I delay acting upon it
   1) Very seldom or not true of me
   2) Rarely true of me
   3) Sometimes true of me
   4) Often true of me
   5) Very often true or true of me

5. I waste a lot of time on trivial matters before getting to the final decisions
   1) Very seldom or not true of me
   2) Rarely true of me
   3) Sometimes true of me
   4) Often true of me
   5) Very often true or true of me

6. In preparation for some deadlines, I often waste time by doing other things
   1) Very seldom or not true of me
   2) Rarely true of me
   3) Sometimes true of me
   4) Often true of me
   5) Very often true or true of me

7. Even jobs that require little else except sitting down and doing them, I find that they seldom get done for days
   1) Very seldom or not true of me
   2) Rarely true of me
   3) Sometimes true of me
   4) Often true of me
   5) Very often true or true of me

8. I often find myself performing tasks that I had intended to do days before
   1) Very seldom or not true of me
   2) Rarely true of me
   3) Sometimes true of me
   4) Often true of me
   5) Very often true or true of me

9. I am continually saying ”I´ll do it tomorrow”
   1) Very seldom or not true of me
   2) Rarely true of me
   3) Sometimes true of me
   4) Often true of me
   5) Very often true or true of me

10. I generally delay before starting on work I have to do
   1) Very seldom or not true of me
   2) Rarely true of me
   3) Sometimes true of me
   4) Often true of me
   5) Very often true or true of me

11. I find myself running out of time
   1) Very seldom or not true of me
   2) Rarely true of me
   3) Sometimes true of me
   4) Often true of me
   5) Very often true or true of me

12. I don´t get things done on time
   1) Very seldom or not true of me
   2) Rarely true of me
   3) Sometimes true of me
   4) Often true of me
   5) Very often true or true of me

13. I am not very good at meeting deadlines
   1) Very seldom or not true of me
   2) Rarely true of me
   3) Sometimes true of me
   4) Often true of me
   5) Very often true or true of me

14. Putting things off till the last minute has cost me money in the past
   1) Very seldom or not true of me
   2) Rarely true of me
   3) Sometimes true of me
   4) Often true of me
   5) Very often true or true of me

2-MONTH FOLLOW-UP

PROCRASTINATION

1. I delay making decisions until it´s too late
   1) Very seldom or not true of me
   2) Rarely true of me
   3) Sometimes true of me
   4) Often true of me
   5) Very often true or true of me

2. Even after I make a decision I delay acting upon it
   1) Very seldom or not true of me
   2) Rarely true of me
   3) Sometimes true of me
   4) Often true of me
   5) Very often true or true of me

3. I waste a lot of time on trivial matters before getting to the final decisions
   1) Very seldom or not true of me
   2) Rarely true of me
   3) Sometimes true of me
   4) Often true of me
   5) Very often true or true of me

4. In preparation for some deadlines, I often waste time by doing other things
   1) Very seldom or not true of me
   2) Rarely true of me
   3) Sometimes true of me
   4) Often true of me
   5) Very often true or true of me

5. Even jobs that require little else except sitting down and doing them, I find that they seldom get done for days
   1) Very seldom or not true of me
   2) Rarely true of me
   3) Sometimes true of me
   4) Often true of me
   5) Very often true or true of me

6. I often find myself performing tasks that I had intended to do days before
   1) Very seldom or not true of me
   2) Rarely true of me
   3) Sometimes true of me
   4) Often true of me
   5) Very often true or true of me

7. I am continually saying ”I´ll do it tomorrow”
   1) Very seldom or not true of me
   2) Rarely true of me
   3) Sometimes true of me
   4) Often true of me
   5) Very often true or true of me

8. I generally delay before starting on work I have to do
    1) Very seldom or not true of me
    2) Rarely true of me
    3) Sometimes true of me
    4) Often true of me
    5) Very often true or true of me

9. I find myself running out of time
    1) Very seldom or not true of me
    2) Rarely true of me
    3) Sometimes true of me
    4) Often true of me
    5) Very often true or true of me

10. I don´t get things done on time
    1) Very seldom or not true of me
    2) Rarely true of me
    3) Sometimes true of me
    4) Often true of me
    5) Very often true or true of me

11. I am not very good at meeting deadlines
    1) Very seldom or not true of me
    2) Rarely true of me
    3) Sometimes true of me
    4) Often true of me
    5) Very often true or true of me

12. Putting things off till the last minute has cost me money in the past
    1) Very seldom or not true of me
    2) Rarely true of me
    3) Sometimes true of me
    4) Often true of me
    5) Very often true or true of me

ANXIETY AND STRESS

13. Over the last 2 weeks, how often have you been bothered by the following problems?
    1) Feeling nervous, anxious or on the edge
    2) Not being able to stop or control worrying
    3) Worrying too much about different things
    4) Trouble relaxing
    5) Being so restless that it is hard to sit still
    6) Becoming easily annoyed or irritable
    7) Feeling afraid as if something awful might happen
        a. Not at all
        b. Several days
        c. More than half of the days
        d. Nearly every day

14. In the last month, how often have you felt that you were unable to control the important things in your life?
    a. Never
    b. Almost never
    c. Sometimes
    d. Fairly often
    e. Very often

15. In the last month, how often have you felt confident about your ability to handle your personal problems?
    a. Never
    b. Almost never
    c. Sometimes
    d. Fairly often
    e. Very often

16. In the last month, how often have you felt that things were going your way?
    a. Never
    b. Almost never
    c. Sometimes
    d. Fairly often
    e. Very often

17. In the last month, how often have you felt difficulties were piling up so high that you could not overcome them?
    a. Never
    b. Almost never
    c. Sometimes
    d. Fairly often
    e. Very often

LIFESTYLE BEHAVIOURS

Note: Participants are reminded of the definition of a standard unit of alcohol by graphical means, as well as given visual cues for what constitutes a unit of sugary drinks.

18. How many standard drinks of alcohol did you consume last week? (numerical measure)

19. How often, during the past month, have you consumed four or more standard drinks of alcohol on one occasion? (numerical measure)

20. How many cans (33 cl, one standard can) of sugary drinks (e.g. soft drinks, “energy drinks”) did you consume last week?

    a. 0 cans
    b. 1 can per week
    c. 2-3 cans per week
    d. 4-6 cans per week
    e. 1 can per day
    f. 1.5 cans per day
    g. 2.0 cans per day
    h. 2.5 cans per day
    i. 3.0 cans per day or more

21. How many portions of candy, chocolate, pastry (e.g. buns, muffins, cookies), ice cream and salty snacks (e.g. crisps, nuts, chees doodles) did you eat last week? *One portion is 50 g candy (9 pieces), 40 g chocolate (6 pieces/squares), 1 bun, 2 dl (scoops) of ice cream or 2 dl snacks (40 g)*.

a. 0 portions
b. 1 portion per week
c. 2-3 portions per week
d. 4-6 portions per week
e. 1 portion per day
f. 1.5 portions per day
g. 2.0 portions per day
h. 2.5 portions per day
i. 3.0 portions per day
j. 3.5 portions per day
k. 4.0 portions per day or more

22. How much time in total did you spend on moderate physical activity (e.g. bicycling or walking for transport or leisure) last week?

a. 0
b. Less than 30 minutes
c. 30-60 minutes
d. 1 hours
e. 1.5 hours
f. 2 hours
g. 2.5 hours
h. 3 hours
i. 3.5 hours (i.e. 30 minutes per day)
j. 4 hours
k. 5 hours
l. 6 hours
m. 7 hours (i.e. 1 hour per day)
n. 10.5 hours (i.e. 1.5 hours per day)
o. 14 hours (i.e. 2 hours per day)

23. How much time in total did you spend on vigorous physical activity (i.e. producing fast increases in breathing or heart rate), for instance running, aerobics, etc. last week?

a. 0
b. Less than 30 minutes
c. 30-60 minutes
d. 1 hours
e. 1.5 hours
f. 2 hours
g. 2.5 hours
h. 3 hours
i. 3.5 hours (i.e. 30 minutes per day)
j. 4 hours
k. 5 hours
l. 6 hours
m. 7 hours (i.e. 1 hour per day)
n. 10.5 hours (i.e. 1.5 hours per day)
o. 14 hours (i.e. 2 hours per day)

PERCEIVED USEFULNESS AND GENERAL OPINION OF THE SUPPORT RECEIVED

24. Overall, how well did the support given to you suit your needs?
    (1 = “Not very helpful” to 5 = “Very helpful”)

25. Please leave a comment describing your needs and how the support did or did not address them (Free-text).

26. Do you believe that the support given to you would be helpful for other individuals that want to change their procrastination behaviour?
    (1 = “Not very helpful” to 5 = “Very helpful”)
