# Supplementary material for: Evaluating the effectiveness of a brief digital procrastination intervention targeting university students in Sweden: study protocol for the Focus randomised controlled trial
Source: BMJ Open. 2023 Jul 21;13(7):e072506. doi: 10.1136/bmjopen-2023-072506 (PMC10364182; doi:10.1136/bmjopen-2023-072506)
Supplement: Author's manuscript [file bmjopen-2023-072506.draft_revisions.pdf]

BMJ Open is committed to open peer review. As part of this commitment we make the peer review history of every article we publish publicly available.

When an article is published we post the peer reviewers' comments and the authors' responses online. We also post the versions of the paper that were used during peer review. These are the versions that the peer review comments apply to.

The versions of the paper that follow are the versions that were submitted during the peer review process. They are not the versions of record or the final published versions. They should not be cited or distributed as the published version of this manuscript.

BMJ Open is an open access journal and the full, final, typeset and author-corrected version of record of the manuscript is available on our site with no access controls, subscription charges or pay-per-view fees (http://bmjopen.bmj.com).

If you have any questions on BMJ Open's open peer review process please email info.bmjopen@bmj.com

BMJ Open

Evaluating the effectiveness of a brief digital procrastination intervention targeting university students in Sweden: study protocol for the Focus randomised controlled trial

Journal:	*BMJ Open*
Manuscript ID	bmjopen-2023-072506
Article Type:	Protocol
Date Submitted by the Author:	07-Feb-2023
Complete List of Authors:	Åsberg, Katarina; Linköping University, Department of Health, Medicine and Caring Sciences<br>Bendtsen, Marcus; Linköping University, Department of Health, Medicine and Caring Sciences
Keywords:	PREVENTIVE MEDICINE, Primary Prevention, PUBLIC HEALTH

SCHOLARONE™
Manuscripts

Evaluating the effectiveness of a brief digital procrastination intervention targeting university students in Sweden: study protocol for the *Focus* randomised controlled trial

Katarina Åsberg[1,*], Marcus Bendtsen[1]

[1] Department of Health, Medicine and Caring Sciences, Linköping University, Sweden.

*Corresponding author
Katarina Åsberg (katarina.asberg@liu.se)
Department of Health, Medicine and Caring Sciences, Division of Society and Health
Linköping University, 581 83 Linköping, registrator@liu.se, +46 13 28 10 00

For peer review only

For peer review only - http://bmjopen.bmj.com/site/about/guidelines.xhtml

Abstract

Introduction: The concept of procrastination can be described as a conscious, yet irrational, postponement of important tasks or decisions - despite awareness that the delay may lead to negative consequences. Procrastination behaviours are common among university students and is often described as a failure of self-regulation, and the behaviour is associated with stress, symptoms of depression and anxiety, poorer academic performance, and negative effects on overall health and wellbeing.

Methods and analysis: A two-arm, parallel groups (1:1), single blind randomised controlled trial will be conducted to assess the effectiveness of a brief digital procrastination intervention on procrastination among university students in Sweden. The primary outcome will be procrastination behaviour measured at 2-months post-randomisation. Secondary outcomes will be anxiety and stress symptoms and lifestyle behaviours. Outcomes will be analysed using multilevel regression models estimated using Bayesian inference.

Ethics and dissemination: The study was approved by the Swedish Ethical Review Authority on 2022-08-24 (dnr 2022-00353). Recruitment will begin in February 2023. A final dataset will be available by the end of April 2023, and findings will be submitted for peer review in open access journals in 2024.

Trial registration number: This trial has been prospectively registered on 2023-02-03 (ISRCTN13533793).

Keywords: public health, mHealth, health behaviour change, procrastination, university students.

Article summary

Strengths and limitations of this study

- Previous research on procrastination has mainly focused on examining mechanisms behind the behaviour, and to a lesser extent on evaluating support that could be helpful in changing procrastination behaviours.
- The effectiveness of a novel brief digital intervention aiming to help college and university students change their procrastination behaviours will be evaluated.
- The trial design necessitates self-assessment of behaviours (self-monitoring), which itself may influence behaviours, leaving a potential risk of bias since the control group will be offered a chance to self-assess their behaviours.
- Attrition is a concern in digital intervention research and is anticipated to be a source of bias in the trial.

For peer review only

Introduction

The concept of procrastination can be described as a conscious, yet irrational, postponement of important tasks or decisions - despite awareness that the delay may lead to negative consequences. To procrastinate has been defined by Steel as "to voluntarily delay an intended course of action despite expecting to be worse off for the delay" [1]. For example, procrastinators tend to voluntarily keep busy with unrelated tasks instead of working to meet deadlines, leading to a short-term relief of undesired feelings associated with the prioritised task.

Procrastination is often described as a failure of self-regulation [2], and the behaviour is associated with stress, symptoms of depression and anxiety [3,4], poorer academic performance [5], and negative effect on overall health and wellbeing [6]. Previous research on procrastination has mainly focused on examining mechanisms behind the behaviour, for example why people procrastinate and associations with personality traits such as impulsivity [7], and to a lesser extent on evaluating support that could be helpful in changing procrastination behaviours [8]. Although procrastination of health behaviours is relatively unexplored in the literature [9], two examples of domain specific procrastination are 'bedtime procrastination' leading to insufficient sleep [10], and 'exercise procrastination' associated with lower overall physical activity even after controlling for intentions and general procrastination [11].

Procrastination behaviours are common among university students and previous studies indicate that approximately 50% of students are likely to procrastinate their academic assignments or tasks [12,13]. Also, in the general population, procrastination tends to be more common among younger adults aged 14-29 years [4]. For many students, the transition to higher education involves more academic demands and lesser external control of things such as scheduled hours versus attention competition from social and digital activities. Students are responsible for their own study technique, and previously used strategies such as 'last-minute-task performance' might create a breeding ground for procrastination, which in turn might end up in negative health consequences.

The majority of scientifically evaluated procrastination support have been based on cognitive behaviour therapy (CBT) treatment programs, delivered both in face-to-face and digital settings, and both with and without additional support from a therapist [14–19]. For example, a study by Rozental et al. [14] evaluated guided and unguided self-help using internet based CBT-treatment in the general population and showed moderate effect on procrastination. Another study from Rozental et al. compared internet based CBT with group therapy among university students with severe procrastination symptoms, and showed no statistically significant differences between groups after treatment [15]. In 2018, Eckert et al. [16] evaluated a two-week unguided online intervention for university students to overcome procrastination, with and without additional text messages, which showed small to medium effects on procrastination.

Since procrastination is common among university students, and since it may lead to ill-health and have a negative impact on academic performance, there is a need to develop and evaluate a scalable, brief digital procrastination intervention to reach a general student population in need of support.

For peer review only - http://bmjopen.bmj.com/site/about/guidelines.xhtml

Aims and objectives

The primary aim of this study, which is called *Focus*, is to estimate the effects of a brief digital procrastination intervention on procrastination behaviours among university students in Sweden. Secondary aims include estimation of the effects of the intervention on anxiety and stress symptoms, as well as lifestyle behaviours. The study objectives are to:

1. Estimate the effects of a brief digital procrastination intervention on procrastination behaviours.
2. Estimate the effects of a brief digital procrastination intervention on anxiety and stress symptoms.
3. Estimate the effects of a brief digital procrastination intervention on alcohol consumption, intake of candy and snacks, sugary drinks, and physical activity.
4. Investigate acceptability, perceived usefulness, and general opinions of the intervention in terms of user experiences.

Methods

A two-arm, parallel groups (1:1), single blind randomised controlled trial (RCT) will be conducted to assess the effectiveness of a brief digital procrastination intervention targeting university students. This protocol includes relevant items from the Standard Protocol Items: Recommendations for Interventional Trials (SPIRIT) 2013 statement [20].

Study setting, recruitment, and eligibility

The Focus study will be situated at colleges and universities in Sweden. Students at participating colleges and universities will be invited via email by their university student health care to participate in a study, in which they are asked to assess their procrastination. The email contains a hyperlink to a webpage presenting study information and informed consent materials (see Appendix A). Students who give informed consent will immediately be taken to a web-based questionnaire which will collect baseline data and check for eligibility.

Eligibility criteria

Students completing the baseline questionnaire and scoring 20 points or more on the Pure Procrastination Scale (PPS) will be eligible for randomisation into the trial. As the survey will be conducted in Swedish, students not familiar with the Swedish language will be implicitly excluded. Excluded students will be given access to the digital intervention but will not be randomised into the study and not followed-up. After completing the questionnaire, eligible participants will be randomised to one of the two conditions: the Focus intervention (intervention group) or a non-treatment condition (control group). Figure 1 outlines the study design.

Insert Fig. 1 here.

For peer review only - http://bmjopen.bmj.com/site/about/guidelines.xhtml

Figure 1. Flowchart of study design

Interventions

Summary score and information (control group)

Participants allocated to the control group will be shown their total PPS score, with the minimum and maximum on the scale presented, and a recommendation to read more about procrastination at their local student health care website.

The Focus intervention (intervention group)

Participants allocated to the intervention group will be given the same information as the control group, but also be given access to the novel Focus intervention.

The Focus intervention is a digital procrastination intervention which consists of a screening and feedback component, allowing participants to assess their current procrastination behaviours and receive behaviour change advice. The intervention is designed with inspiration from both previous research on procrastination treatment programs using internet-based cognitive behavioural therapy (iCBT) [14,15], and on the concept of brief alcohol interventions, which have been widely researched among university students in Sweden [21].

The intervention was designed in iterative steps following the first stage ('understanding the behaviour') of the Behaviour Change Wheel (BCW) as a theoretical framework [22,23]. Procrastination behaviour was understood by defining the problem in behavioural terms, specifying the target behaviour, and identifying what needs to change in dialogue with professionals at the student health care centres during several multidisciplinary group meetings. Intervention content was also designed and developed in collaboration with the student health care centres.

The intervention starts with participants assessing their procrastination behaviour using the PPS questionnaire. Following the assessment, participants are given feedback on their current procrastination behaviours and personalised behaviour change advice. The items of the PPS have been found to load onto a 3-factor model [24] where items 1-3 relate to decisional delay, items 4-8 to implemental delay, and items 9-12 to timeliness and promptness delay. The feedback and advice given to participants as part of the digital intervention is therefore tailored in accordance with these latent constructs, allowing for prioritisation of advice based on highest scoring construct.

The personalised advice component is based on the self-assessment and consists of graphical, written, and interactive content. The feedback aims to enhance self-reflection and self-evaluation and aims to stimulate self-reinforcement upon participants procrastination behaviour, in line with self-regulation theory [25]. Figure 2 visualises screenshots of the intervention.

Insert Fig. 2 here.

Figure 2. Screenshots of the intervention

For peer review only - http://bmjopen.bmj.com/site/about/guidelines.xhtml

Outcomes

The following outcome measures will be used. All study questionnaires can be found in Appendix B.

Primary outcome measures:

- Procrastination behaviour.

Secondary outcome measures:

- Anxiety.
- Stress.
- Weekly alcohol consumption.
- Monthly frequency of heavy episodic drinking.
- Weekly consumption of candy and snacks.
- Weekly consumption of sugary drinks.
- Weekly moderate to vigorous physical activity (MVPA).

User evaluation:

- Perceived acceptability and, usefulness of the intervention among users.

Primary and secondary outcome measures

Procrastination behaviour will be assessed by asking participants to complete the PPS 12-item Pure Procrastination Scale. Each item is scored on a five-point Likert scale with higher score indicating greater difficulties with procrastination (12-60). The scale was originally developed by Steel [26], and has been translated to Swedish and validated by Rozental et al. [27], showing good internal consistency (Cronbach´s a=0.78).

Anxiety will be assessed using the validated seven item Generalised Anxiety Disorder scale (GAD-7) [28], asking participants how often they, over the last two weeks, have been bothered by seven different anxiety related problems. Perceived stress will be assessed using the four item short form perceived stress scale (PSS-4), designed to measure experienced levels of stress [29], and considered a useful instrument for assessing stress perception levels in the general population [30].

Weekly alcohol consumption will be assessed by asking participants the number of standard drinks of alcohol they consumed last week (short term recall method). Frequency of heavy episodic drinking will be assessed by asking participants how many times they have consumed 4 or more standard drinks of alcohol on one occasion the past month. These two outcomes are both part of the proposed core outcome set for brief alcohol interventions [31].

For peer review only - http://bmjopen.bmj.com/site/about/guidelines.xhtml

Diet and physical activity will be measured utilising a questionnaire based on the previously published questionnaire designed by the National Board of Health and Welfare in Sweden, and was further modified to also include portion sizes [32]. Candy and snacks will be measured using a single question regarding number of servings consumed last week, and sugary drinks consumption will be measured by a question regarding the number of units (33 cl) of sugary drinks participants consumed the past week. Moderate and vigorous physical activity will be estimated by summing responses to two questions regarding the number of minutes spent on moderate and vigorous physical activity, respectively, during the past week.

At two-months follow-up, participants allocated to the intervention group will be asked three questions about perceived acceptability and usefulness of the intervention.

Participant timeline and follow-ups

Recruitment, informed consent, eligibility screening, randomisation, baseline assessment, intervention, and follow-ups will all be automated and computerised. Two email-reminders will be sent one week apart. Follow-up at two months after randomisation will be initiated by sending an email with a hyperlink to a web-based questionnaire. Non-responders to follow-up will receive up to three follow-up email reminders. A study timeline is presented in Figure 3.

Insert Fig. 3 here.

Figure 3. SPIRIT figure presenting participant timeline.

Assignment of interventions

Participants will be randomised using block randomisation (with random block sizes of 2 and 4). The randomisation sequence will be computer generated, and neither research personnel nor participants will be able to manipulate the sequence. As all study procedures are automated, thus randomisation cannot be subverted.

Sample size

The data collected in this trial will be analysed using Bayesian analysis [33], which delivers a posterior probability of effect regardless of the number of participants included. We will not use null hypothesis testing; therefore, a traditional power calculation is not required to determine the required sample size to control type I and II errors. Instead, we conducted a Monte Carlo simulation study, assuming a standardised effect of 0.35 Cohen's d of the intervention on the primary outcome (procrastination behaviour). To at least 80% of the time estimate a posterior probability of effect of at least 95.0%, the Monte Carlo simulations indicated that data from 1 000 participants are required for analysis, meaning that 2 000 participants are required to be randomised when assuming a 50% attrition rate.

Data analysis

All analyses will be conducted keeping participants in the groups to which they were randomised (intention-to-treat). We will conduct available data analyses and use multiple imputation with chained equations (with 200 imputed data sets using 30 iterations) to impute missing data for imputed analyses. Both available data and imputed analyses will be used to interpret findings. All models will be estimated using Bayesian inference, with the posterior probability of effect being

For peer review only - http://bmjopen.bmj.com/site/about/guidelines.xhtml

presented along with posterior medians and 95% compatibility intervals. All models will include an adaptive intercept for each university (multilevel), with standard normal priors.

The primary outcome (PPS) will be standardised and analysed using linear regression, adjusted for age, gender, and PPS at baseline. Anxiety, stress, weekly consumption of candy and snacks, and MVPA will be modelled equivalently as PPS. Frequency of heavy episodic drinking, weekly alcohol consumption, and weekly consumption of sugary drinks will be analysed using negative binomial regression, adjusted for age, gender and PPS at baseline. Student-t priors will be used for covariate coefficients, normal priors will be used for adaptive intercepts with half-Student-t hyper-priors for the standard deviation, and half-Student-t priors will also be used for main standard deviations and dispersion parameters. All Student-t priors will have 3 degrees of freedom and a scale of 2.5.

Interaction analyses will be conducted between treatment allocation and age, gender, and baseline PPS. Models will be compared using the Widely Applicable Information Criterion (WAIC) to decide if interaction models are informative in comparison to non-interaction models.

Attrition analyses will be conducted to investigate systematic differences between responders and non-responders. Logistic regression will be used to estimate the odds ratio of not responding to follow-up with respect to age, gender, and baseline PPS. The same priors as the primary analyses will be used.

Patient and Public Involvement

Previous research, interviewing university students about their experiences of health and health-related behaviours highlighted procrastination and study-related stress as a common barrier for not engaging in healthy routines such as physical activity and healthy cooking [34]. This study was designed and developed in collaboration with professionals at student health care centres across Sweden. During the process of designing the intervention content and usability testing of the digital support, approximately ten professionals took part in multidisciplinary group meetings during 2022. These meetings defined procrastination in behavioural terms, specified the target behaviour, identified what needs to change, and ways to achieve this using a digital tool.

Discussion

This randomised controlled trial aims to estimate the effects of a brief digital procrastination intervention on procrastination behaviours among university students in Sweden. It will add findings on how to support students to overcome procrastination - something that is inadequately evaluated. This is so despite procrastination behaviours being common among university students, and known to cause ill-health, distress and negatively impact academic performance [3,5]. Digital interventions could fulfil these needs and are scalable to a large group of individuals at a low cost. The low cost of delivering digital interventions [35], and the low participant burden of completing a brief screening questionnaire, justifies even small effects from the intervention.

Generalisability

This study is carried out in a college and university setting, reaching students via communication channels used in their academic and daily lives. Students signing up to participate in the study do not represent a population consisting entirely of individuals actively seeking help, and the inclusion

For peer review only - http://bmjopen.bmj.com/site/about/guidelines.xhtml

criteria are relatively broad for inclusion. The study can therefore be understood as an effectiveness trial, which aims to estimate the effects of the intervention in a in a naturalistic setting.

Limitations

The trial design necessitates self-assessment of procrastination behaviours among all participants prior to randomisation. Previous research has shown that assessment of behaviours (self-monitoring) may have an effect on self-reported behaviour [36,37], thus there is risk of diluting effects by the control group being offered a chance to self-assess their procrastination behaviours. This is however unavoidable in order to maintain the naturalistic design of the study and to conduct data collection. Most conservatively, estimated effects may therefore be viewed as caused by the feedback and advice component only, assuming no interaction with self-assessment.

Attrition bias is a concern in digital intervention research, including this study. We are anticipating high attrition at 50%, which is difficult to recover from even using modern methods of imputation. Attrition analyses will reveal if there is evidence of systematic differences among responders and non-responders; however, the study will nevertheless be limited by the risk of attrition bias.

Public health relevance

Procrastinating affects both physical and mental health negatively and can, apart from increased stress, involve risk behaviours, such as excessive alcohol consumption or tobacco use as stress-relieving strategies. Procrastination behaviour might also out-compete good health habits such as sleep hygiene, regular physical activity or healthy eating habits, due to high level of stress. By quitting postponing important commitments students increase the chance of succeeding with their studies, decreasing their risk of mental illness, exhaustion and anxiety-related problems. Today, there is a lack of support to help students take control of their procrastination behaviours. Therefor, and due to the high prevalence of procrastination among students, it is valuable to evaluate a scalable digital procrastination support for university students.

Ethics and dissemination

The study was approved by the Swedish Ethical Review Authority on 2022-08-24 (dnr 2022-00353). Recruitment will begin in February 2023. A final dataset will be available by the end of April 2023, and findings will be submitted for peer review in open access journals in 2024.

Declarations

Contributors: Study objectives, outcomes, trial design and analysis plan were decided by MB and KÅ, as well as development and conceptualisation of intervention materials. KÅ drafted the protocol, which was revised by MB. Both authors contributed with intellectual content and approved the final version. KÅ and MB will be responsible for data collection and statistical analysis.

Competing interests: Author MB owns a private company (Alexit AB) that maintains and distributes evidence-based lifestyle interventions to be used by the public and in health care settings. Alexit AB played no role in developing the intervention, study design, data analysis, data interpretation, or writing of this report. Author KÅ declare no competing interests.

For peer review only - http://bmjopen.bmj.com/site/about/guidelines.xhtml

Funding: This trial is conducted under the auspices of the Swedish Research Council for Health, Working Life, and Welfare (Grant number 2018-01410). The funders had no role in study design, data collection and analysis, decision to publish, or preparation of the manuscript.

Data availability statement: Deidentified datasets generated during and/or analysed during the current study will be made available upon reasonable request to the corresponding author, after approval of a proposal and with a signed data access agreement.

Acknowledgements: We would like to acknowledge the student health care professionals at the participating universities who provided us with their time and shared their experiences.

For peer review only

References

1. Steel P. The nature of procrastination: a meta-analytic and theoretical review of quintessential self-regulatory failure. *Psychol Bull*. 2007;133(1):65-94. doi:10.1037/0033-2909.133.1.65, PMID:17201571
2. Fernie BA, Bharucha Z, Nikčević A V, Marino C, Spada MM. A Metacognitive model of procrastination. *J Affect Disord*. 2017;210:196-203. doi:10.1016/j.jad.2016.12.042, PMID:28056361
3. Johansson F, Rozental A, Edlund K, Côté P, Sundberg T, Onell C, Rudman A, Skillgate E. Associations Between Procrastination and Subsequent Health Outcomes Among University Students in Sweden. *JAMA Netw open*. 2023;6(1):e2249346. doi:10.1001/jamanetworkopen.2022.49346, PMID:36598789
4. Beutel ME, Klein EM, Aufenanger S, Brähler E, Dreier M, Müller KW, Quiring O, Reinecke L, Schmutzer G, Stark B, Wölfling K. Procrastination, Distress and Life Satisfaction across the Age Range - A German Representative Community Study. *PLoS One*. 2016;11(2):e0148054. doi:10.1371/journal.pone.0148054, PMID:26871572
5. Garzón-Umerenkova A, de la Fuente J, Amate J, Paoloni P V, Fadda S, Pérez JF. A Linear Empirical Model of Self-Regulation on Flourishing, Health, Procrastination, and Achievement, Among University Students. *Front Psychol*. 2018;9:536. doi:10.3389/fpsyg.2018.00536, PMID:29706922
6. Basirimoghadam M, Rafii F, Ebadi A. Self-rated health and general procrastination in nurses: a cross-sectional study. *Pan Afr Med J*. 2020;36:254. doi:10.11604/pamj.2020.36.254.23720, PMID:33014250
7. Zhang S, Liu P, Feng T. To do it now or later: The cognitive mechanisms and neural substrates underlying procrastination. *Wiley Interdiscip Rev Cogn Sci*. 2019;10(4):e1492. doi:10.1002/wcs.1492, PMID:30638308
8. Zacks S, Hen M. Academic interventions for academic procrastination: A review of the literature. *J Prev Interv Community*. 2018;46(2):117-130. doi:10.1080/10852352.2016.1198154, PMID:29485384
9. Kroese FM, de Ridder DTD. Health behaviour procrastination: a novel reasoned route towards self-regulatory failure. *Health Psychol Rev*. 2016;10(3):313-325. doi:10.1080/17437199.2015.1116019, PMID:26530737
10. Kroese FM, Evers C, Adriaanse MA, de Ridder DTD. Bedtime procrastination: A self-regulation perspective on sleep insufficiency in the general population. *J Health Psychol*. 2016;21(5):853-862. doi:10.1177/1359105314540014, PMID:24997168
11. Kelly SM, Walton HR. “I’ll work out tomorrow”: The Procrastination in Exercise Scale. *J Health Psychol*. 2021;26(13):2613-2625. doi:10.1177/1359105320916541, PMID:32459106
12. Atalayin C, Balkis M, Tezel H, Kayrak G. Procrastination and predictor variables among a group of dental students in Turkey. *Psychol Health Med*. 2018;23(6):726-732. doi:10.1080/13548506.2017.1418014, PMID:29262718
13. Ferrari JR, Ozer BU, Demir A. Chronic procrastination among Turkish adults: exploring decisional, avoidant, and arousal styles. *J Soc Psychol*. 2009;149(3):402-408. doi:10.3200/SOCP.149.3.402-408, PMID:19537606
14. Rozental A, Forsell E, Svensson A, Andersson G, Carlbring P. Internet-based cognitive-behavior therapy for procrastination: A randomized controlled trial. *J Consult Clin Psychol*. 2015;83(4):808-824. doi:10.1037/ccp0000023, PMID:25939016
15. Rozental A, Forsström D, Lindner P, Nilsson S, Mårtensson L, Rizzo A, Andersson G, Carlbring P. Treating Procrastination Using Cognitive Behavior Therapy: A Pragmatic Randomized Controlled Trial Comparing Treatment Delivered via the Internet or in Groups. *Behav Ther*. 2018;49(2):180-197. doi:10.1016/j.beth.2017.08.002, PMID:29530258
16. Eckert M, Ebert DD, Lehr D, Sieland B, Berking M. Does SMS-Support Make a Difference? Effectiveness of a Two-Week Online-Training to Overcome Procrastination. A Randomized

For peer review only - http://bmjopen.bmj.com/site/about/guidelines.xhtml

Controlled Trial. *Front Psychol*. 2018;9:1103. doi:10.3389/fpsyg.2018.01103, PMID:30026713
17. Gagnon J, Dionne F, Raymond G, Grégoire S. Pilot study of a Web-based acceptance and commitment therapy intervention for university students to reduce academic procrastination. *J Am Coll Health*. 2019;67(4):374-382. doi:10.1080/07448481.2018.1484361, PMID:29979942
18. Lukas CA, Berking M. Reducing procrastination using a smartphone-based treatment program: A randomized controlled pilot study. *Internet Interv*. 2018;12:83-90. doi:10.1016/j.invent.2017.07.002, PMID:30135772
19. Küchler A-M, Albus P, Ebert DD, Baumeister H. Effectiveness of an internet-based intervention for procrastination in college students (StudiCare Procrastination): Study protocol of a randomized controlled trial. *Internet Interv*. 2019;17:100245. doi:10.1016/j.invent.2019.100245, PMID:31080750
20. Chan A-W, Tetzlaff JM, Altman DG, Laupacis A, Gøtzsche PC, Krleža-Jerić K, Hróbjartsson A, Mann H, Dickersin K, Berlin JA, Doré CJ, Parulekar WR, Summerskill WSM, Groves T, Schulz KF, Sox HC, Rockhold FW, Rennie D, Moher D. SPIRIT 2013 statement: defining standard protocol items for clinical trials. *Ann Intern Med*. 2013;158(3):200-207. doi:10.7326/0003-4819-158-3-201302050-00583, PMID:23295957
21. Bendtsen P, McCambridge J, Bendtsen M, Karlsson N, Nilsen P. Effectiveness of a proactive mail-based alcohol Internet intervention for university students: dismantling the assessment and feedback components in a randomized controlled trial. *J Med Internet Res*. 2012;14(5):e142. doi:10.2196/jmir.2062, PMID:23113955
22. Michie, S.; Atkins, L.; West R. *The Behaviour Change Wheel: A Guide to Designing Interventions*. UK: Silverback publishing; 2015. http://www.behaviourchangewheel.com/.
23. Michie S, van Stralen MM, West R. The behaviour change wheel: a new method for characterising and designing behaviour change interventions. *Implement Sci*. 2011;6:42. doi:10.1186/1748-5908-6-42, PMID:21513547
24. Svartdal F, Steel P. Irrational Delay Revisited: Examining Five Procrastination Scales in a Global Sample. *Front Psychol*. 2017;8:1927. doi:10.3389/fpsyg.2017.01927, PMID:29163302
25. Kanfer FH, Gaelick-Buys L. Self-management methods. In: *Helping People Change: A Textbook of Methods, 4th Ed.* Pergamon general psychology series, Vol. 52. Elmsford, NY, US: Pergamon Press; 1991:305-360.
26. Steel P. Arousal, avoidant and decisional procrastinators: Do they exist? *Pers Individ Dif*. 2010;48(8):926-934. doi:https://doi.org/10.1016/j.paid.2010.02.025
27. Rozental A, Forsell E, Svensson A, Forsström D, Andersson G, Carlbring P. Psychometric evaluation of the Swedish version of the pure procrastination scale, the irrational procrastination scale, and the susceptibility to temptation scale in a clinical population. *BMC Psychol*. 2014;2(1):54. doi:10.1186/s40359-014-0054-z, PMID:25566392
28. Spitzer RL, Kroenke K, Williams JBW, Löwe B. A brief measure for assessing generalized anxiety disorder: the GAD-7. *Arch Intern Med*. 2006;166(10):1092-1097. doi:10.1001/archinte.166.10.1092, PMID:16717171
29. Cohen S, Kamarck T, Mermelstein R. A Global Measure of Perceived Stress. *J Health Soc Behav*. 1983;24(4):385-396. http://www.jstor.org/stable/2136404. Accessed June 13, 2022.
30. Vallejo MA, Vallejo-Slocker L, Fernández-Abascal EG, Mañanes G. Determining factors for stress perception assessed with the Perceived Stress Scale (PSS-4) in Spanish and other European samples. *Front Psychol*. 2018;9(JAN). doi:10.3389/fpsyg.2018.00037
31. Shorter GW, Bray JW, Heather N, Berman AH, Giles EL, Clarke M, Barbosa C, O'Donnell AJ, Holloway A, Riper H, Daeppen J-B, Monteiro MG, Saitz R, McNeely J, McKnight-Eily L, Cowell A, Toner P, Newbury-Birch D. The "Outcome Reporting in Brief Intervention Trials: Alcohol" (ORBITAL) Core Outcome Set: International Consensus on Outcomes to Measure in Efficacy and Effectiveness Trials of Alcohol Brief Interventions. *J Stud Alcohol Drugs*. 2021;82(5):638-646. doi:10.15288/jsad.2021.82.638, PMID:34546911
32. Socialstyrelsen. *Nationella Riktlinjer För Prevention Och Behandling Vid Ohälsosamma*

For peer review only - http://bmjopen.bmj.com/site/about/guidelines.xhtml

Levnadsvanor.; 2018. https://www.socialstyrelsen.se/globalassets/sharepoint-dokument/artikelkatalog/nationella-riktlinjer/2018-6-24.pdf.

33. Bendtsen M. A Gentle Introduction to the Comparison Between Null Hypothesis Testing and Bayesian Analysis: Reanalysis of Two Randomized Controlled Trials. *J Med Internet Res*. 2018;20(10):e10873. doi:10.2196/10873
34. Åsberg K, Eldh AC, Löf M, Bendtsen M. A balancing act-finding one´s way to health and well-being: A qualitative analysis of interviews with Swedish university students on lifestyle and behavior change. *PLoS One*. 2022;17(10):e0275848. doi:10.1371/journal.pone.0275848, PMID:36227904
35. Ebert JF, Huibers L, Christensen B, Christensen MB. Paper- or Web-Based Questionnaire Invitations as a Method for Data Collection: Cross-Sectional Comparative Study of Differences in Response Rate, Completeness of Data, and Financial Cost. *J Med Internet Res*. 2018;20(1):e24. doi:10.2196/jmir.8353, PMID:29362206
36. McCambridge J, Bendtsen M, Karlsson N, White IR, Nilsen P, Bendtsen P. Alcohol assessment and feedback by email for university students: main findings from a randomised controlled trial. *Br J Psychiatry*. 2013;203(5):334-340. doi:10.1192/bjp.bp.113.128660, PMID:24072758
37. Bendtsen M, McCambridge J. Causal models accounted for research participation effects when estimating effects in a behavioral intervention trial. *J Clin Epidemiol*. 2021;136:77-83. doi:10.1016/j.jclinepi.2021.03.008, PMID:33727133

For peer review only

For peer review only - http://bmjopen.bmj.com/site/about/guidelines.xhtml

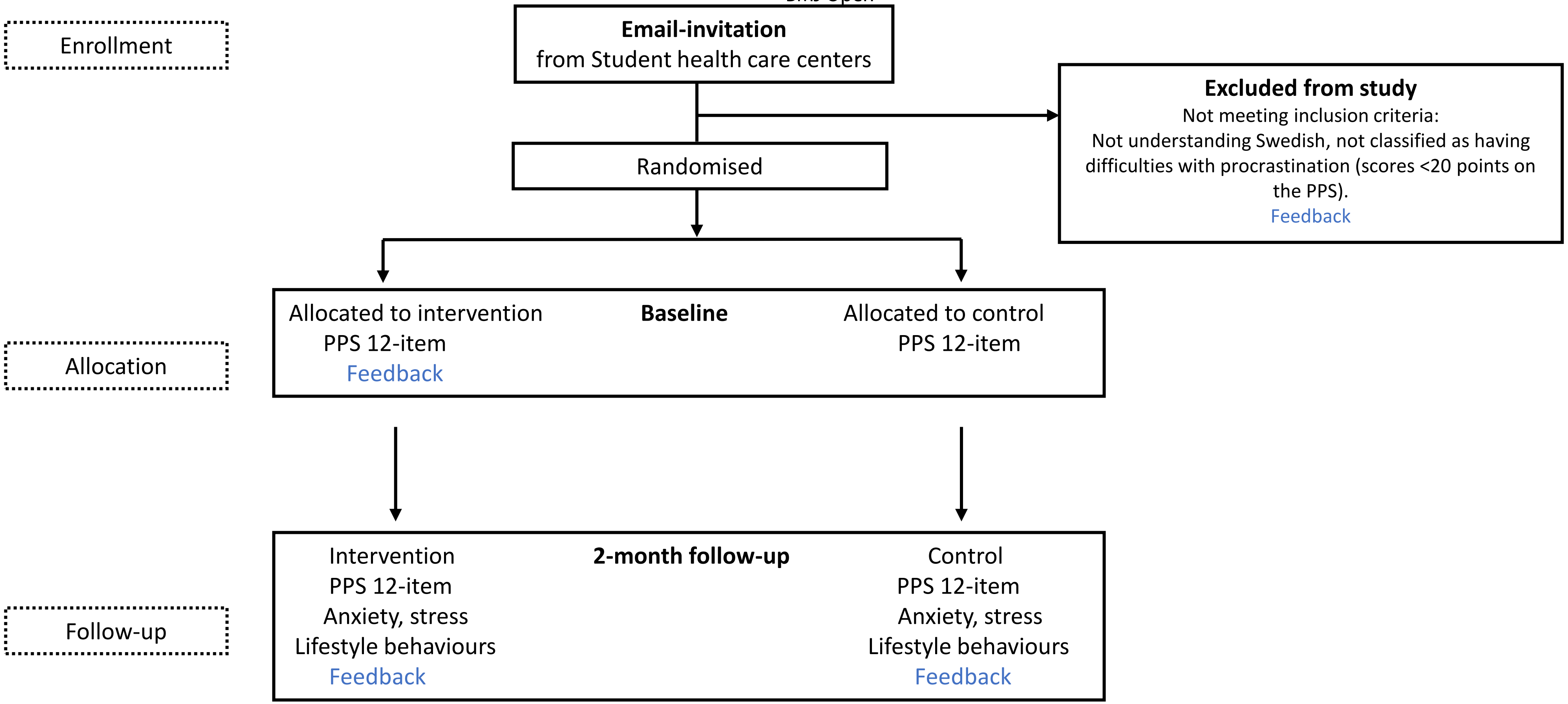
Enrollment
Email-invitation
from Student health care centers
Excluded from study
Not meeting inclusion criteria:
Not understanding Swedish, not classified as having difficulties with procrastination (scores <20 points on the PPS).
Feedback
Randomised
Allocation
Allocated to intervention
PPS 12-item
Feedback
Baseline
Allocated to control
PPS 12-item
Follow-up
Intervention
PPS 12-item
Anxiety, stress
Lifestyle behaviours
Feedback
2-month follow-up
Control
PPS 12-item
Anxiety, stress
Lifestyle behaviours
Feedback

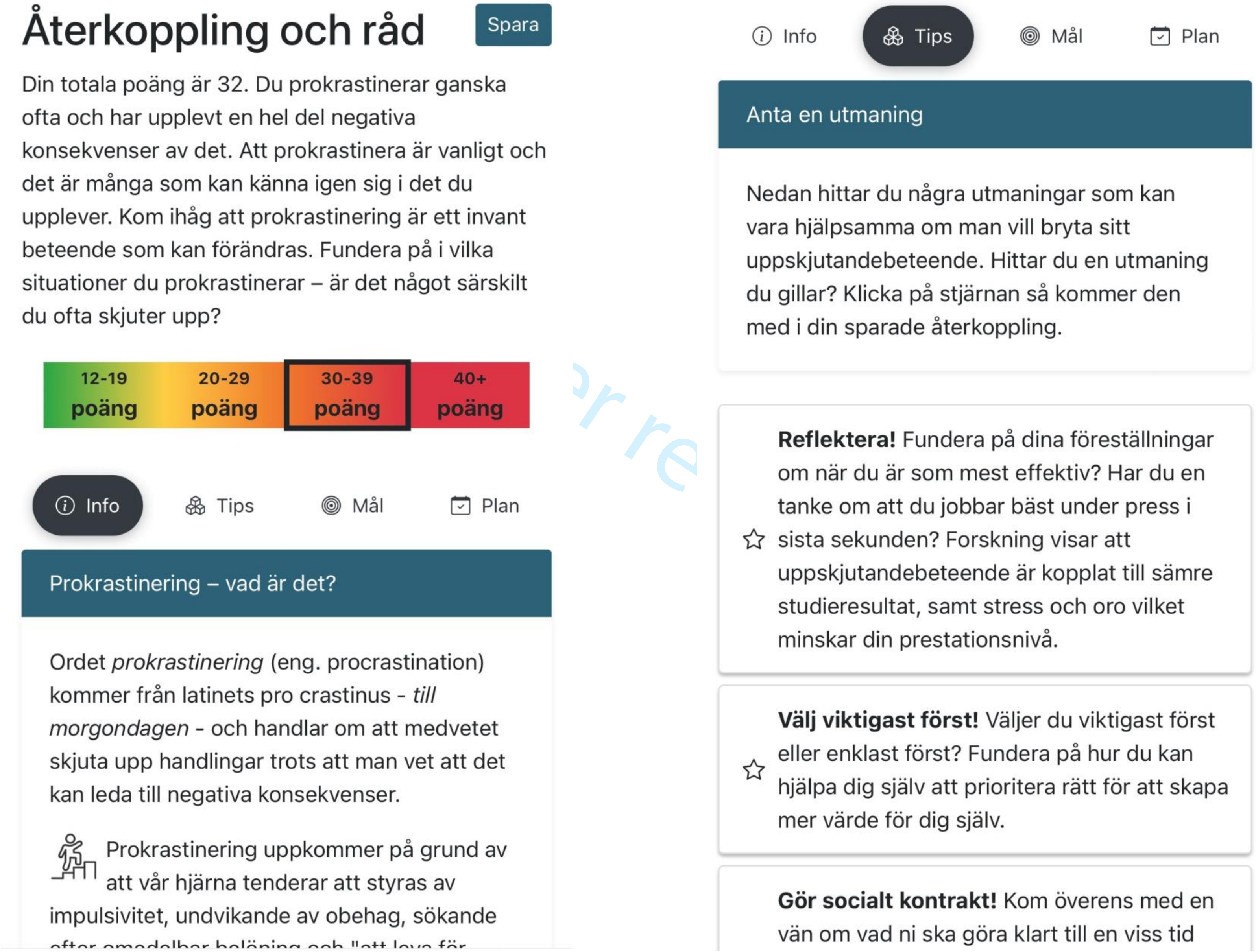
Återkoppling och råd
Spara
Din totala poäng är 32. Du prokrastinerar ganska ofta och har upplevt en hel del negativa konsekvenser av det. Att prokrastinera är vanligt och det är många som kan känna igen sig i det du upplever. Kom ihåg att prokrastinering är ett invant beteende som kan förändras. Fundera på i vilka situationer du prokrastinerar – är det något särskilt du ofta skjuter upp?
12-19 poäng
20-29 poäng
30-39 poäng
40+ poäng
Info
Tips
Mål
Plan
Prokrastinering – vad är det?
Ordet prokrastinering (eng. procrastination) kommer från latinets pro crastinus - till morgondagen - och handlar om att medvetet skjuta upp handlingar trots att man vet att det kan leda till negativa konsekvenser.
Prokrastinering uppkommer på grund av att vår hjärna tenderar att styras av impulsivitet, undvikande av obehag, sökande
Info
Tips
Mål
Plan
Anta en utmaning
Nedan hittar du några utmaningar som kan vara hjälpsamma om man vill bryta sitt uppskjutandebeteende. Hittar du en utmaning du gillar? Klicka på stjärnan så kommer den med i din sparade återkoppling.
Reflektera! Fundera på dina föreställningar om när du är som mest effektiv? Har du en tanke om att du jobbar bäst under press i sista sekunden? Forskning visar att uppskjutandebeteende är kopplat till sämre studieresultat, samt stress och oro vilket minskar din prestationsnivå.
Välj viktigast först! Väljer du viktigast först eller enklast först? Fundera på hur du kan hjälpa dig själv att prioritera rätt för att skapa mer värde för dig själv.
Gör socialt kontrakt! Kom överens med en vän om vad ni ska göra klart till en viss tid

Figure. SPIRIT figure presenting participant timeline for the Focus procrastination study

	STUDY PERIOD			
	**Enrolment**	**Allocation**	**Post-allocation**	**Close-out**
**TIMEPOINT**	**0**	**0**	**0**	***2 months***
**ENROLMENT:**				
**Informed consent**	X			
**Eligibility screen**	X			
**Allocation**		X		
**INTERVENTIONS:**				
Digital intervention		X ⟶	⟶	⟶
Control condition		X ⟶	⟶	⟶
**ASSESSMENTS:**				
Baseline questionnaire	X			
Pure procrastination scale	X			X
Lifestyle questionnaire				X
User evaluation questionnaire				X

For peer review only

APPENDIX A – INFORMED CONSENT

We would like to ask you to participate in a research project. On this page you can find information about the project and what participation entails.

WHY ARE WE DOING THIS STUDY AND WHY DO YOU WANT ME TO PARTICIPATE?

Focus is a research project which aims to estimate the effects of a brief digital procrastination intervention on procrastination, anxiety and stress symptoms, and lifestyle behaviours among university students in Sweden. The intervention is delivered online and since you are a college or university student enrolled at a university in Sweden, we would like to ask you if you would like to participate in the study.

The research project is conducted at Linköping University in Sweden, please find contact details for the primary investigator below.

WHAT IS EXPECTED OF ME?

Those who consent to take part in the study will first be asked to complete a short, online questionnaire about their current levels of procrastination. Participants will thereafter receive feedback on their current level of procrastination. Depending on which version of Focus you have been assigned to, you might also receive access to resources designed to support you change your procrastination behaviour. After two months, all participants will, again, be asked to complete a follow-up questionnaire regarding their current levels of anxiety and stress, procrastination, and lifestyle behaviours (via e-mail). The questionnaires take 5-10 minutes to complete.

WILL I BE TAKING ANY RISKS BY PARTICIPATING IN THIS STUDY?

If you decide to take part in the study then you should be aware that while Focus has been designed based on current scientific evidence regarding how to support individuals to change their procrastination behaviours, however, not everyone who uses Focus will succeed in procrastinating less. This may feel like a failure and may be de-motivating. Participants should also be aware that changing behaviours and challenging oneself to act differently, may initially result in discomfort and increased stress. These discomforts are passing, and in the long run the benefits of a more sustainable study situation will outweigh the discomforts.

You can always contact your student- or primary-healthcare centre if you feel any discomforts, want more help, or wish to consult a professional regarding your mental or physical health. You can also contact 1177 if you have any questions about your health.

WILL I BE LEAVING ANY PERSONAL INFORMATION?

The project will collect and register information about you. No unauthorized persons will have access to your answers.

Your responses to the questionnaires during the study period will be stored in a database at Linköping University. We will use an encrypted version of your e-mail address in order to connect your responses from the different questionnaires. The secret key used to decrypt your e-mail address will only be accessible by the primary investigator Marcus Bendtsen (see contact information below). Neither during analysis nor publication of findings will any information be connected to you individually.

For peer review only

When the project is complete, all e-mail addresses will be deleted. The information will be stored at Linköping University for 10 years. The data collection is for scientific research and is therefore motivated by public interest (GDPR EU 2016/679, Prop. 2017/18:298).

Linköping University is responsible for your data. According to EU:s data protection regulation you have the right to, free of charge, be given access to the data collected about you in the project, and have errors corrected. You may also request that your data be deleted or use of your data be restricted. However, the right to erasure and to limit the processing of personal data does not apply when the data is necessary for the current research. If you wish to be given access to your data, you should contact the primary investigator Marcus Bendtsen (see contact information below). The data protection officer can be reached at dataskyddsombud@liu.se. If you are dissatisfied with the way your personal data has been handled, you can file a complaint at the Swedish Authority for Privacy Protection.

HOW CAN I GET MORE INFORMATION ABOUT THE RESULTS FROM THIS STUDY?

After the study has been completed, findings will be published in scientific peer-reviewed journals. Findings at the individual level will not be traceable from these publications. We will not contact you after study completion, but you are welcome to contact us if you want information on findings or copies of published reports.

INSURANCE

As a participant of a research project at Linköping University you are included in the insurance the university has at Kammarkollegiet.

PARTICIPATION IS FREE

Participation is free and you can at any time decide to end it. If you decide to end your participation you will not be asked why, and it will not affect your studies. If you wish to end your participation you should contact the primary investigator Marcus Bendtsen (see contact information below).

PRIMARY INVESTIGATOR

Marcus Bendtsen, PhD
Docent in Medical Informatics
Senior Lecturer in Experimental Social Medicine and Public Health
Department of Health Medicine and Caring Sciences, Linköping University
marcus.bendtsen@liu.se, 013-286975

For peer review only

APPENDIX B – QUESTIONNAIRES

BASELINE

1. Sex:
    a. Female
    b. Male
    c. Nonbinary
    d. Other

2. Age (numerical measure)

PROCRASTINATION

3. I delay making decisions until it´s too late
    1) Very seldom or not true of me
    2) Rarely true of me
    3) Sometimes true of me
    4) Often true of me
    5) Very often true or true of me

4. Even after I make a decision I delay acting upon it
    1) Very seldom or not true of me
    2) Rarely true of me
    3) Sometimes true of me
    4) Often true of me
    5) Very often true or true of me

5. I waste a lot of time on trivial matters before getting to the final decisions
    1) Very seldom or not true of me
    2) Rarely true of me
    3) Sometimes true of me
    4) Often true of me
    5) Very often true or true of me

6. In preparation for some deadlines, I often waste time by doing other things
    1) Very seldom or not true of me
    2) Rarely true of me
    3) Sometimes true of me
    4) Often true of me
    5) Very often true or true of me

7. Even jobs that require little else except sitting down and doing them, I find that they seldom get done for days
    1) Very seldom or not true of me
    2) Rarely true of me
    3) Sometimes true of me
    4) Often true of me
    5) Very often true or true of me

For peer review only

8. I often find myself performing tasks that I had intended to do days before
    1) Very seldom or not true of me
    2) Rarely true of me
    3) Sometimes true of me
    4) Often true of me
    5) Very often true or true of me

9. I am continually saying ”I´ll do it tomorrow”
    1) Very seldom or not true of me
    2) Rarely true of me
    3) Sometimes true of me
    4) Often true of me
    5) Very often true or true of me

10. I generally delay before starting on work I have to do
    1) Very seldom or not true of me
    2) Rarely true of me
    3) Sometimes true of me
    4) Often true of me
    5) Very often true or true of me

11. I find myself running out of time
    1) Very seldom or not true of me
    2) Rarely true of me
    3) Sometimes true of me
    4) Often true of me
    5) Very often true or true of me

12. I don´t get things done on time
    1) Very seldom or not true of me
    2) Rarely true of me
    3) Sometimes true of me
    4) Often true of me
    5) Very often true or true of me

13. I am not very good at meeting deadlines
    1) Very seldom or not true of me
    2) Rarely true of me
    3) Sometimes true of me
    4) Often true of me
    5) Very often true or true of me

14. Putting things off till the last minute has cost me money in the past
    1) Very seldom or not true of me
    2) Rarely true of me
    3) Sometimes true of me
    4) Often true of me
    5) Very often true or true of me

For peer review only

2-MONTH FOLLOW-UP

PROCRASTINATION

1. I delay making decisions until it´s too late
   1) Very seldom or not true of me
   2) Rarely true of me
   3) Sometimes true of me
   4) Often true of me
   5) Very often true or true of me

2. Even after I make a decision I delay acting upon it
   1) Very seldom or not true of me
   2) Rarely true of me
   3) Sometimes true of me
   4) Often true of me
   5) Very often true or true of me

3. I waste a lot of time on trivial matters before getting to the final decisions
   1) Very seldom or not true of me
   2) Rarely true of me
   3) Sometimes true of me
   4) Often true of me
   5) Very often true or true of me

4. In preparation for some deadlines, I often waste time by doing other things
   1) Very seldom or not true of me
   2) Rarely true of me
   3) Sometimes true of me
   4) Often true of me
   5) Very often true or true of me

5. Even jobs that require little else except sitting down and doing them, I find that they seldom get done for days
   1) Very seldom or not true of me
   2) Rarely true of me
   3) Sometimes true of me
   4) Often true of me
   5) Very often true or true of me

6. I often find myself performing tasks that I had intended to do days before
   1) Very seldom or not true of me
   2) Rarely true of me
   3) Sometimes true of me
   4) Often true of me
   5) Very often true or true of me

7. I am continually saying ”I´ll do it tomorrow”
   1) Very seldom or not true of me
   2) Rarely true of me
   3) Sometimes true of me
   4) Often true of me
   5) Very often true or true of me

For peer review only

8. I generally delay before starting on work I have to do
   1) Very seldom or not true of me
   2) Rarely true of me
   3) Sometimes true of me
   4) Often true of me
   5) Very often true or true of me

9. I find myself running out of time
   1) Very seldom or not true of me
   2) Rarely true of me
   3) Sometimes true of me
   4) Often true of me
   5) Very often true or true of me

10. I don´t get things done on time
   1) Very seldom or not true of me
   2) Rarely true of me
   3) Sometimes true of me
   4) Often true of me
   5) Very often true or true of me

11. I am not very good at meeting deadlines
   1) Very seldom or not true of me
   2) Rarely true of me
   3) Sometimes true of me
   4) Often true of me
   5) Very often true or true of me

12. Putting things off till the last minute has cost me money in the past
   1) Very seldom or not true of me
   2) Rarely true of me
   3) Sometimes true of me
   4) Often true of me
   5) Very often true or true of me

ANXIETY AND STRESS

13. Over the last 2 weeks, how often have you been bothered by the following problems?
   1) Feeling nervous, anxious or on the edge
   2) Not being able to stop or control worrying
   3) Worrying too much about different things
   4) Trouble relaxing
   5) Being so restless that it is hard to sit still
   6) Becoming easily annoyed or irritable
   7) Feeling afraid as if something awful might happen
      a. Not at all
      b. Several days
      c. More than half of the days
      d. Nearly every day

For peer review only

14. In the last month, how often have you felt that you were unable to control the important things in your life?
   a. Never
   b. Almost never
   c. Sometimes
   d. Fairly often
   e. Very often

15. In the last month, how often have you felt confident about your ability to handle your personal problems?
   a. Never
   b. Almost never
   c. Sometimes
   d. Fairly often
   e. Very often

16. In the last month, how often have you felt that things were going your way?
   a. Never
   b. Almost never
   c. Sometimes
   d. Fairly often
   e. Very often

17. In the last month, how often have you felt difficulties were piling up so high that you could not overcome them?
   a. Never
   b. Almost never
   c. Sometimes
   d. Fairly often
   e. Very often

LIFESTYLE BEHAVIOURS

Note: Participants are reminded of the definition of a standard unit of alcohol by graphical means, as well as given visual cues for what constitutes a unit of sugary drinks.

18. How many standard drinks of alcohol did you consume last week? (numerical measure)

19. How often, during the past month, have you consumed four or more standard drinks of alcohol on one occasion? (numerical measure)

20. How many cans (33 cl, one standard can) of sugary drinks (e.g. soft drinks, "energy drinks") did you consume last week?

   a. 0 cans
   b. 1 can per week
   c. 2-3 cans per week
   d. 4-6 cans per week
   e. 1 can per day
   f. 1.5 cans per day
   g. 2.0 cans per day
   h. 2.5 cans per day
   i. 3.0 cans per day or more

For peer review only

21. How many portions of candy, chocolate, pastry (e.g. buns, muffins, cookies), ice cream and salty snacks (e.g. crisps, nuts, chees doodles) did you eat last week? *One portion is 50 g candy (9 pieces), 40 g chocolate (6 pieces/squares), 1 bun, 2 dl (scoops) of ice cream or 2 dl snacks (40 g)*.

a. 0 portions
b. 1 portion per week
c. 2-3 portions per week
d. 4-6 portions per week
e. 1 portion per day
f. 1.5 portions per day
g. 2.0 portions per day
h. 2.5 portions per day
i. 3.0 portions per day
j. 3.5 portions per day
k. 4.0 portions per day or more

22. How much time in total did you spend on moderate physical activity (e.g. bicycling or walking for transport or leisure) last week?

a. 0
b. Less than 30 minutes
c. 30-60 minutes
d. 1 hours
e. 1.5 hours
f. 2 hours
g. 2.5 hours
h. 3 hours
i. 3.5 hours (i.e. 30 minutes per day)
j. 4 hours
k. 5 hours
l. 6 hours
m. 7 hours (i.e. 1 hour per day)
n. 10.5 hours (i.e. 1.5 hours per day)
o. 14 hours (i.e. 2 hours per day)

23. How much time in total did you spend on vigorous physical activity (i.e. producing fast increases in breathing or heart rate), for instance running, aerobics, etc. last week?

a. 0
b. Less than 30 minutes
c. 30-60 minutes
d. 1 hours
e. 1.5 hours
f. 2 hours
g. 2.5 hours
h. 3 hours
i. 3.5 hours (i.e. 30 minutes per day)
j. 4 hours
k. 5 hours
l. 6 hours
m. 7 hours (i.e. 1 hour per day)
n. 10.5 hours (i.e. 1.5 hours per day)
o. 14 hours (i.e. 2 hours per day)

For peer review only

PERCEIVED USEFULNESS AND GENERAL OPINION OF THE SUPPORT RECEIVED

24. Overall, how well did the support given to you suit your needs?
    (1 = "Not very helpful" to 5 = "Very helpful")

25. Please leave a comment describing your needs and how the support did or did not address them (Free-text).

26. Do you believe that the support given to you would be helpful for other individuals that want to change their procrastination behaviour?
    (1 = "Not very helpful" to 5 = "Very helpful")

For peer review only

STANDARD PROTOCOL ITEMS: RECOMMENDATIONS FOR INTERVENTIONAL TRIALS

SPIRIT 2013 Checklist: Recommended items to address in a clinical trial protocol and related documents*

Section/item	Item No	Description	Addressed on page number
**Administrative information**			
Title	1	Descriptive title identifying the study design, population, interventions, and, if applicable, trial acronym	______1______
Trial registration	2a	Trial identifier and registry name. If not yet registered, name of intended registry	_____1,2______
	2b	All items from the World Health Organization Trial Registration Data Set	_____1,2______
Protocol version	3	Date and version identifier	______NA____
Funding	4	Sources and types of financial, material, and other support	_____ 12_____
Roles and responsibilities	5a	Names, affiliations, and roles of protocol contributors	_____ 1______
	5b	Name and contact information for the trial sponsor	_____12____
	5c	Role of study sponsor and funders, if any, in study design; collection, management, analysis, and interpretation of data; writing of the report; and the decision to submit the report for publication, including whether they will have ultimate authority over any of these activities	_____12____
	5d	Composition, roles, and responsibilities of the coordinating centre, steering committee, endpoint adjudication committee, data management team, and other individuals or groups overseeing the trial, if applicable (see Item 21a for data monitoring committee)	_____NA_____

For peer review only

Introduction

Background and rationale	6a	Description of research question and justification for undertaking the trial, including summary of relevant studies (published and unpublished) examining benefits and harms for each intervention	_____3______
	6b	Explanation for choice of comparators	_____3, 4, 5_____
Objectives	7	Specific objectives or hypotheses	_____4_______
Trial design	8	Description of trial design including type of trial (eg, parallel group, crossover, factorial, single group), allocation ratio, and framework (eg, superiority, equivalence, noninferiority, exploratory)	_____4______

Methods: Participants, interventions, and outcomes

Study setting	9	Description of study settings (eg, community clinic, academic hospital) and list of countries where data will be collected. Reference to where list of study sites can be obtained	_____4_______
Eligibility criteria	10	Inclusion and exclusion criteria for participants. If applicable, eligibility criteria for study centres and individuals who will perform the interventions (eg, surgeons, psychotherapists)	_____4______
Interventions	11a	Interventions for each group with sufficient detail to allow replication, including how and when they will be administered	____4,5_______
	11b	Criteria for discontinuing or modifying allocated interventions for a given trial participant (eg, drug dose change in response to harms, participant request, or improving/worsening disease)	_____NA______
	11c	Strategies to improve adherence to intervention protocols, and any procedures for monitoring adherence (eg, drug tablet return, laboratory tests)	_____NA_____
	11d	Relevant concomitant care and interventions that are permitted or prohibited during the trial	_____NA______
Outcomes	12	Primary, secondary, and other outcomes, including the specific measurement variable (eg, systolic blood pressure), analysis metric (eg, change from baseline, final value, time to event), method of aggregation (eg, median, proportion), and time point for each outcome. Explanation of the clinical relevance of chosen efficacy and harm outcomes is strongly recommended	_____6,7______
Participant timeline	13	Time schedule of enrolment, interventions (including any run-ins and washouts), assessments, and visits for participants. A schematic diagram is highly recommended (see Figure)	__7, Figure 3__

For peer review only - http://bmjopen.bmj.com/site/about/guidelines.xhtml

Sample size	14	Estimated number of participants needed to achieve study objectives and how it was determined, including clinical and statistical assumptions supporting any sample size calculations	_____7______
Recruitment	15	Strategies for achieving adequate participant enrolment to reach target sample size	_____7,8______

Methods: Assignment of interventions (for controlled trials)

Allocation:

Sequence generation	16a	Method of generating the allocation sequence (eg, computer-generated random numbers), and list of any factors for stratification. To reduce predictability of a random sequence, details of any planned restriction (eg, blocking) should be provided in a separate document that is unavailable to those who enrol participants or assign interventions	______8_______
Allocation concealment mechanism	16b	Mechanism of implementing the allocation sequence (eg, central telephone; sequentially numbered, opaque, sealed envelopes), describing any steps to conceal the sequence until interventions are assigned	_____8________
Implementation	16c	Who will generate the allocation sequence, who will enrol participants, and who will assign participants to interventions	_____8________
Blinding (masking)	17a	Who will be blinded after assignment to interventions (eg, trial participants, care providers, outcome assessors, data analysts), and how	_____8________
	17b	If blinded, circumstances under which unblinding is permissible, and procedure for revealing a participant's allocated intervention during the trial	_____8________

Methods: Data collection, management, and analysis

Data collection methods	18a	Plans for assessment and collection of outcome, baseline, and other trial data, including any related processes to promote data quality (eg, duplicate measurements, training of assessors) and a description of study instruments (eg, questionnaires, laboratory tests) along with their reliability and validity, if known. Reference to where data collection forms can be found, if not in the protocol	____4,7________
	18b	Plans to promote participant retention and complete follow-up, including list of any outcome data to be collected for participants who discontinue or deviate from intervention protocols	_____7,8_______

Data management	19	Plans for data entry, coding, security, and storage, including any related processes to promote data quality (eg, double data entry; range checks for data values). Reference to where details of data management procedures can be found, if not in the protocol	___NA_________
Statistical methods	20a	Statistical methods for analysing primary and secondary outcomes. Reference to where other details of the statistical analysis plan can be found, if not in the protocol	____8,9________
	20b	Methods for any additional analyses (eg, subgroup and adjusted analyses)	____8,9_______
	20c	Definition of analysis population relating to protocol non-adherence (eg, as randomised analysis), and any statistical methods to handle missing data (eg, multiple imputation)	____8,9_______
**Methods: Monitoring**			
Data monitoring	21a	Composition of data monitoring committee (DMC); summary of its role and reporting structure; statement of whether it is independent from the sponsor and competing interests; and reference to where further details about its charter can be found, if not in the protocol. Alternatively, an explanation of why a DMC is not needed	_____NA______
	21b	Description of any interim analyses and stopping guidelines, including who will have access to these interim results and make the final decision to terminate the trial	_____NA______
Harms	22	Plans for collecting, assessing, reporting, and managing solicited and spontaneously reported adverse events and other unintended effects of trial interventions or trial conduct	_____NA_______
Auditing	23	Frequency and procedures for auditing trial conduct, if any, and whether the process will be independent from investigators and the sponsor	_____NA_______
**Ethics and dissemination**			
Research ethics approval	24	Plans for seeking research ethics committee/institutional review board (REC/IRB) approval	______2,9_____
Protocol amendments	25	Plans for communicating important protocol modifications (eg, changes to eligibility criteria, outcomes, analyses) to relevant parties (eg, investigators, REC/IRBs, trial participants, trial registries, journals, regulators)	______NA_____

Consent or assent	26a	Who will obtain informed consent or assent from potential trial participants or authorised surrogates, and how (see Item 32)	______4, App A___
	26b	Additional consent provisions for collection and use of participant data and biological specimens in ancillary studies, if applicable	______N/A______
Confidentiality	27	How personal information about potential and enrolled participants will be collected, shared, and maintained in order to protect confidentiality before, during, and after the trial	______App A______
Declaration of interests	28	Financial and other competing interests for principal investigators for the overall trial and each study site	______12______
Access to data	29	Statement of who will have access to the final trial dataset, and disclosure of contractual agreements that limit such access for investigators	______12________
Ancillary and post-trial care	30	Provisions, if any, for ancillary and post-trial care, and for compensation to those who suffer harm from trial participation	______NA_______
Dissemination policy	31a	Plans for investigators and sponsor to communicate trial results to participants, healthcare professionals, the public, and other relevant groups (eg, via publication, reporting in results databases, or other data sharing arrangements), including any publication restrictions	______ 9, App A__
	31b	Authorship eligibility guidelines and any intended use of professional writers	______12_______
	31c	Plans, if any, for granting public access to the full protocol, participant-level dataset, and statistical code	______ 12 ______
**Appendices**			
Informed consent materials	32	Model consent form and other related documentation given to participants and authorised surrogates	______App A___
Biological specimens	33	Plans for collection, laboratory evaluation, and storage of biological specimens for genetic or molecular analysis in the current trial and for future use in ancillary studies, if applicable	______NA_______

*It is strongly recommended that this checklist be read in conjunction with the SPIRIT 2013 Explanation & Elaboration for important clarification on the items. Amendments to the protocol should be tracked and dated. The SPIRIT checklist is copyrighted by the SPIRIT Group under the Creative Commons "Attribution-NonCommercial-NoDerivs 3.0 Unported" license.

For peer review only - http://bmjopen.bmj.com/site/about/guidelines.xhtml

BMJ Open

Evaluating the effectiveness of a brief digital procrastination intervention targeting university students in Sweden: study protocol for the Focus randomised controlled trial

Journal:	*BMJ Open*
Manuscript ID	bmjopen-2023-072506.R1
Article Type:	Protocol
Date Submitted by the Author:	02-Jun-2023
Complete List of Authors:	Åsberg, Katarina; Linköping University, Department of Health, Medicine and Caring Sciences<br>Bendtsen, Marcus; Linköping University, Department of Health, Medicine and Caring Sciences
**Primary Subject Heading**:	Public health
Secondary Subject Heading:	Public health
Keywords:	PREVENTIVE MEDICINE, Primary Prevention, PUBLIC HEALTH

SCHOLARONE™
Manuscripts

Evaluating the effectiveness of a brief digital procrastination intervention targeting university students in Sweden: study protocol for the *Focus* randomised controlled trial

Katarina Åsberg[1,*], Marcus Bendtsen[1]

[1] Department of Health, Medicine and Caring Sciences, Linköping University, Sweden.

*Correspondence to:
Katarina Åsberg (katarina.asberg@liu.se)
Department of Health, Medicine and Caring Sciences, Division of Society and Health
Linköping University, 581 83 Linköping, registrator@liu.se, +46 13 28 10 00

For peer review only

For peer review only - http://bmjopen.bmj.com/site/about/guidelines.xhtml

Abstract

Introduction: The concept of procrastination can be described as a conscious, yet irrational, postponement of important tasks or decisions - despite awareness that the delay may lead to negative consequences. Procrastination behaviours are common among university students and is often described as a failure of self-regulation, and the behaviour is associated with stress, symptoms of depression and anxiety, poorer academic performance, and negative effects on overall health and wellbeing.

Methods and analysis: A two-arm, parallel groups (1:1), single blind randomised controlled trial will be conducted to assess the effectiveness of a brief digital procrastination intervention (Focus) among university students in Sweden. The intervention consists of a screening and feedback component based on Pure Procrastination Scale (PPS) score, allowing intervention participants to assess their current procrastination behaviours and receive behaviour change advice. Participants in the control group will be shown their total PPS score without any further feedback. Monte Carlo simulations (assuming a standardised effect of 0.35 Cohen's d of the intervention on the primary outcome, to at least 80% of the time estimate a posterior probability of effect of at least 95%) indicated that data from 1 000 participants are required for analysis, meaning that 2 000 participants are required to be randomised when assuming a 50% attrition rate. The primary outcome will be procrastination behaviour measured at 2-months post-randomisation. Secondary outcomes will be anxiety and stress symptoms and lifestyle behaviours. Outcomes will be analysed using multilevel regression models estimated using Bayesian inference.

Ethics and dissemination: The study was approved by the Swedish Ethical Review Authority on 2022-08-24 (dnr 2022-00353). Students will be asked to give informed consent before participation and after having been given information about the study. The results will be submitted for publication in open access, peer-reviewed journals.

Trial registration: Prospectively registered on 2023-02-03, ISRCTN13533793.

Keywords: public health, mHealth, health behaviour change, procrastination, university students.

For peer review only

Article summary

Strengths and limitations of this study

- Previous research on procrastination has mainly focused on examining mechanisms behind the behaviour, and to a lesser extent on evaluating support that could be helpful in changing procrastination behaviours.
- The effectiveness of a novel brief digital intervention aiming to help college and university students change their procrastination behaviours will be evaluated.
- The trial design necessitates self-assessment of behaviours (self-monitoring), which itself may influence behaviours, leaving a potential risk of bias since the control group will be offered a chance to self-assess their behaviours.

For peer review only - http://bmjopen.bmj.com/site/about/guidelines.xhtml

- Attrition is a concern in digital intervention research and is anticipated to be a source of bias in the trial.

For peer review only

For peer review only - http://bmjopen.bmj.com/site/about/guidelines.xhtml

Introduction

The concept of procrastination can be described as a conscious, yet irrational, postponement of important tasks or decisions - despite awareness that the delay may lead to negative consequences. To procrastinate has been defined by Steel as “to voluntarily delay an intended course of action despite expecting to be worse off for the delay” [1]. For example, procrastinators tend to voluntarily keep busy with unrelated tasks instead of working to meet deadlines, leading to a short-term relief of undesired feelings associated with the prioritised task.

Procrastination is often described as a failure of self-regulation [2], and the behaviour is associated with stress, symptoms of depression and anxiety [3,4], poorer academic performance [5], and negative effect on overall health and wellbeing [6]. Previous research on procrastination has mainly focused on examining mechanisms behind the behaviour, for example why people procrastinate and associations with personality traits such as impulsivity [7], and to a lesser extent on evaluating support that could be helpful in changing procrastination behaviours [8]. Although procrastination of health behaviours is relatively unexplored in the literature [9], two examples of domain specific procrastination are ‘bedtime procrastination’ leading to insufficient sleep [10], and ‘exercise procrastination’ associated with lower overall physical activity even after controlling for intentions and general procrastination [11].

Procrastination behaviours are common among university students and previous studies indicate that approximately 50% of students are likely to procrastinate their academic assignments or tasks [12,13]. Also, in the general population, procrastination tends to be more common among younger adults aged 14-29 years [4]. For many students, the transition to higher education involves more academic demands and lesser external control of things such as scheduled hours versus attention competition from social and digital activities. Students are responsible for their own study technique, and previously used strategies such as ‘last-minute-task performance’ might create a breeding ground for procrastination, which in turn might end up in negative health consequences.

The majority of scientifically evaluated procrastination support have been based on cognitive behaviour therapy (CBT) treatment programs, delivered both in face-to-face and digital settings, and both with and without additional support from a therapist [14–19]. For example, a study by Rozental et al. [14] evaluated guided and unguided self-help using internet based CBT-treatment in the general population and showed moderate effect on procrastination. Another study from Rozental et al. compared internet based CBT with group therapy among university students with severe procrastination symptoms, and showed no statistically significant differences between groups after treatment [15]. In 2018, Eckert et al. [16] evaluated a two-week unguided online intervention for university students to overcome procrastination, with and without additional text messages, which showed small to medium effects on procrastination.

Since procrastination is common among university students, and since it may lead to ill-health and have a negative impact on academic performance, there is a need to develop and evaluate a scalable, brief digital procrastination intervention to reach a general student population in need of support.

For peer review only - http://bmjopen.bmj.com/site/about/guidelines.xhtml

Aims and objectives

The primary aim of this study, which is called *Focus*, is to estimate the effects of a brief digital procrastination intervention on procrastination behaviours among university students in Sweden. Secondary aims include estimation of the effects of the intervention on anxiety and stress symptoms, as well as lifestyle behaviours. The study objectives are to:

1. Estimate the effects of a brief digital procrastination intervention on procrastination behaviours.
2. Estimate the effects of a brief digital procrastination intervention on anxiety and stress symptoms.
3. Estimate the effects of a brief digital procrastination intervention on alcohol consumption, intake of candy and snacks, sugary drinks, and physical activity.
4. Investigate acceptability, perceived usefulness, and general opinions of the intervention in terms of user experiences.

Methods and analysis

A two-arm, parallel groups (1:1), single blind randomised controlled trial (RCT) will be conducted to assess the effectiveness of a brief digital procrastination intervention targeting university students. This protocol includes relevant items from the Standard Protocol Items: Recommendations for Interventional Trials (SPIRIT) 2013 statement [20].

Study setting, recruitment, and eligibility

The Focus study will be situated at colleges and universities in Sweden. Students at participating colleges and universities will be invited via email by their university student health care to participate in a study, in which they are asked to assess their procrastination. The email contains a hyperlink to a webpage presenting study information and informed consent materials (see Appendix A). Students who give informed consent will immediately be taken to a web-based questionnaire which will collect baseline data and check for eligibility. Recruitment will begin in February 2023, and a final dataset will be available by the end of the semester of 2023.

Eligibility criteria

Students completing the baseline questionnaire and scoring 20 points or more on the Pure Procrastination Scale (PPS) will be eligible for randomisation into the trial. There are no validated (or recommended) cut-offs of the PPS that indicate which individuals have no procrastination issues, rather, it is a continuous measure of procrastination. However, we did not want to include individuals who scored very low (no procrastination) since they would not be the target group in a future intervention implementation. However, we did want to be inclusive and therefore set the cut-off relatively low at 20 points (note the range of PSS is 12-60), excluding only those who report none or very little procrastination. As the survey will be conducted in Swedish, students not familiar with the

For peer review only - http://bmjopen.bmj.com/site/about/guidelines.xhtml

Swedish language will be implicitly excluded. Excluded students will be given access to the digital intervention but will not be randomised into the study and not followed-up. After completing the questionnaire, eligible participants will be randomised to one of the two conditions: the Focus intervention (intervention group) or a non-treatment condition (control group). Figure 1 outlines the study design.

Interventions

Summary score and information (control group)

Participants allocated to the control group will be shown their total PPS score, with the minimum and maximum on the scale presented, and a recommendation to read more about procrastination at their local student health care website.

The Focus intervention (intervention group)

Participants allocated to the intervention group will be given the same information as the control group, but also be given access to the novel Focus intervention.

The Focus intervention is a digital procrastination intervention which is delivered online to students email and consists of a screening and feedback component, allowing participants to assess their current procrastination behaviours and receive behaviour change advice. The intervention is designed with inspiration from both previous research on procrastination treatment programs using internet-based cognitive behavioural therapy (iCBT) [14,15], and on the concept of brief alcohol interventions, which have been widely researched among university students in Sweden [21].

The intervention was designed in iterative steps following the first stage ('understanding the behaviour') of the Behaviour Change Wheel (BCW) as a theoretical framework [22,23]. Procrastination behaviour was understood by defining the problem in behavioural terms, specifying the target behaviour, and identifying what needs to change in dialogue with professionals at the student health care centres during several multidisciplinary group meetings. Intervention content was also designed and developed in collaboration with the student health care centres.

The intervention starts with participants assessing their procrastination behaviour using the PPS questionnaire. Following the assessment, participants are given feedback on their current procrastination behaviours and personalised behaviour change advice. The items of the PPS have been found to load onto a 3-factor model [24] where items 1-3 relate to decisional delay, items 4-8 to implemental delay, and items 9-12 to timeliness and promptness delay. The feedback and advice given to participants as part of the digital intervention is therefore tailored in accordance with these latent constructs, allowing for prioritisation of advice based on highest scoring construct.

The personalised advice component is based on the self-assessment and consists of graphical, written, and interactive content. The feedback aims to enhance self-reflection and self-evaluation and aims to stimulate self-reinforcement upon participants procrastination behaviour, in line with self-regulation theory [25]. Figure 2 visualises screenshots of the intervention.

Outcomes

The following outcome measures will be used. All study questionnaires can be found in Appendix B.

For peer review only - http://bmjopen.bmj.com/site/about/guidelines.xhtml

Primary outcome measures:

- Procrastination behaviour.

Secondary outcome measures:

- Anxiety.
- Stress.
- Weekly alcohol consumption.
- Monthly frequency of heavy episodic drinking.
- Weekly consumption of candy and snacks.
- Weekly consumption of sugary drinks.
- Weekly moderate to vigorous physical activity (MVPA).

User evaluation:

- Perceived acceptability and, usefulness of the intervention among users.

Primary and secondary outcome measures

Procrastination behaviour will be assessed by asking participants to complete the PPS 12-item Pure Procrastination Scale. Each item is scored on a five-point Likert scale with higher score indicating greater difficulties with procrastination (12-60). The scale was originally developed by Steel [26], and has been translated to Swedish and validated by Rozental et al. [27], showing good internal consistency (Cronbach´s a=0.78).

Anxiety will be assessed using the validated seven item Generalised Anxiety Disorder scale (GAD-7) [28], asking participants how often they, over the last two weeks, have been bothered by seven different anxiety related problems. Perceived stress will be assessed using the four item short form perceived stress scale (PSS-4), designed to measure experienced levels of stress [29], and considered a useful instrument for assessing stress perception levels in the general population [30].

Procrastination behaviours are potentially a barrier for healthy lifestyle behaviours; thus we have added lifestyle behaviour outcome measures to explore this hypothesis. Weekly alcohol consumption will be assessed by asking participants the number of standard drinks of alcohol they consumed last week (short term recall method). Frequency of heavy episodic drinking will be assessed by asking participants how many times they have consumed 4 or more standard drinks of alcohol on one occasion the past month. These two outcomes are both part of the proposed core outcome set for brief alcohol interventions [31].

Diet and physical activity will be measured utilising a questionnaire based on the previously published questionnaire designed by the National Board of Health and Welfare in Sweden, and was further modified to also include portion sizes [32]. Candy and snacks will be measured using a single question regarding number of servings consumed last week, and sugary drinks consumption will be measured by a question regarding the number of units (33 cl) of sugary drinks participants consumed

For peer review only - http://bmjopen.bmj.com/site/about/guidelines.xhtml

the past week. Moderate and vigorous physical activity will be estimated by summing responses to two questions regarding the number of minutes spent on moderate and vigorous physical activity, respectively, during the past week.

At two-months follow-up, participants allocated to the intervention group will be asked three questions about perceived acceptability and usefulness of the intervention.

Participant timeline and follow-ups

Recruitment, informed consent, eligibility screening, randomisation, baseline assessment, intervention, and follow-ups will all be automated and computerised. Two email-reminders will be sent one week apart. Follow-up at two months after randomisation will be initiated by sending an email with a hyperlink to a web-based questionnaire. Non-responders to follow-up will receive up to three follow-up email reminders. A study timeline is presented in Figure 3. Note that this is the first ever effectiveness study of a brief digital intervention for procrastination of its kind, and as such we have no previous studies to guide us on how long intervention effects may persist. We took a pragmatic approach and decided to keep the follow-up interval short enough to stay within the same academic semester, but still enough removed from the intervention itself to decide if there were any lasting effects.

Assignment of interventions

Participants will be randomised using block randomisation (with random block sizes of 2 and 4). The randomisation sequence will be computer generated, and neither research personnel nor participants will be able to manipulate the sequence. As all study procedures are automated, thus randomisation cannot be subverted.

Sample size

The data collected in this trial will be analysed using Bayesian analysis [33], which delivers a posterior probability of effect regardless of the number of participants included. We will not use null hypothesis testing; therefore, a traditional power calculation is not required to determine the required sample size to control type I and II errors. Instead, we conducted a Monte Carlo simulation study, assuming a standardised effect of 0.35 Cohen's d of the intervention on the primary outcome (procrastination behaviour). To at least 80% of the time estimate a posterior probability of effect of at least 95%, the Monte Carlo simulations indicated that data from 1 000 participants are required for analysis, meaning that 2 000 participants are required to be randomised when assuming a 50% attrition rate. Note that this intervention and trial is novel, and as such there are no previous studies to rely on the determination of effect size. However, a previous study comparing guided and unguided self-help using Internet-based cognitive behaviour therapy with a wait-list control observed effects sizes ranging from 0.5 to 0.7 Cohen's d [14]. We anticipate that our brief intervention will be less effective than a full therapy program, and we have thus assumed a small to medium Cohen's d of 0.35.

Data analysis

All analyses will be conducted keeping participants in the groups to which they were randomised (intention-to-treat). We will conduct available data analyses and use multiple imputation with chained equations (with 200 imputed data sets using 30 iterations) to impute missing data for

For peer review only - http://bmjopen.bmj.com/site/about/guidelines.xhtml

imputed analyses. Both available data and imputed analyses will be used to interpret findings. All models will be estimated using Bayesian inference, with the posterior probability of effect being presented along with posterior medians and 95% compatibility intervals. All models will include an adaptive intercept for each university (multilevel), with standard normal priors.

The primary outcome (PPS) will be standardised and analysed using linear regression, adjusted for age, gender, and PPS at baseline. Anxiety, stress, weekly consumption of candy and snacks, and MVPA will be modelled equivalently as PPS. Frequency of heavy episodic drinking, weekly alcohol consumption, and weekly consumption of sugary drinks will be analysed using negative binomial regression, adjusted for age, gender and PPS at baseline. Student-t priors will be used for covariate coefficients, normal priors will be used for adaptive intercepts with half-Student-t hyper-priors for the standard deviation, and half-Student-t priors will also be used for main standard deviations and dispersion parameters. All Student-t priors will have 3 degrees of freedom and a scale of 2.5.

Interaction analyses will be conducted between treatment allocation and age, gender, and baseline PPS. Models will be compared using the Widely Applicable Information Criterion (WAIC) to decide if interaction models are informative in comparison to non-interaction models.

Attrition analyses will be conducted to investigate systematic differences between responders and non-responders. Logistic regression will be used to estimate the odds ratio of not responding to follow-up with respect to age, gender, and baseline PPS. The same priors as the primary analyses will be used.

Patient and public involvement

Previous research, interviewing university students about their experiences of health and health-related behaviours highlighted procrastination and study-related stress as a common barrier for not engaging in healthy routines such as physical activity and healthy cooking [34]. This study was designed and developed in collaboration with professionals at student health care centres across Sweden. During the process of designing the intervention content and usability testing of the digital support, approximately ten professionals took part in multidisciplinary group meetings during 2022. These meetings defined procrastination in behavioural terms, specified the target behaviour, identified what needs to change, and ways to achieve this using a digital tool.

Ethics and dissemination

The study was approved by the Swedish Ethical Review Authority on 2022-08-24 (dnr 2022-00353). Students will be asked to give informed consent before participation and after having been given information about the study. The results will be submitted for publication in open access, peer-reviewed journals.

Discussion

This randomised controlled trial aims to estimate the effects of a brief digital procrastination intervention on procrastination behaviours among university students in Sweden. It will add findings on how to support students to overcome procrastination - something that is inadequately evaluated. This is so despite procrastination behaviours being common among university students, and known to cause ill-health, distress and negatively impact academic performance [3,5]. Digital interventions could fulfil these needs and are scalable to a large group of individuals at a low cost. The low cost of

For peer review only - http://bmjopen.bmj.com/site/about/guidelines.xhtml

delivering digital interventions [35], and the low participant burden of completing a brief screening questionnaire, justifies even small effects from the intervention.

Generalisability

This study is carried out in a college and university setting, reaching students via communication channels used in their academic and daily lives. Students signing up to participate in the study do not represent a population consisting entirely of individuals actively seeking help, and the inclusion criteria are relatively broad for inclusion. The study can therefore be understood as an effectiveness trial, which aims to estimate the effects of the intervention in a in a naturalistic setting. It should be noted that the intervention materials are designed for college and university students, and therefore generalisability outside the target population is not anticipated. Also, being a student in Sweden is not necessarily comparable to being a student in other countries, and so generalisability to other countries may be limited.

Limitations

The trial design necessitates self-assessment of procrastination behaviours among all participants prior to randomisation. Previous research has shown that assessment of behaviours (self-monitoring) may have an effect on self-reported behaviour [36,37], thus there is risk of diluting effects by the control group being offered a chance to self-assess their procrastination behaviours. This is however unavoidable in order to maintain the naturalistic design of the study and to conduct data collection. Most conservatively, estimated effects may therefore be viewed as caused by the feedback and advice component only, assuming no interaction with self-assessment.

Attrition bias is a concern in digital intervention research, including this study. We are anticipating high attrition at 50%, which is difficult to recover from even using modern methods of imputation. Attrition analyses will reveal if there is evidence of systematic differences among responders and non-responders; however, the study will nevertheless be limited by the risk of attrition bias.

Finally, we have chosen a pragmatic follow-up interval of two months. This means that we will not be able to identify earlier effects of the intervention which may have waned.

Public health relevance

Procrastinating affects both physical and mental health negatively and can, apart from increased stress, involve risk behaviours, such as excessive alcohol consumption or tobacco use as stress-relieving strategies. Procrastination behaviour might also out-compete good health habits such as sleep hygiene, regular physical activity or healthy eating habits, due to high level of stress. By quitting postponing important commitments students increase the chance of succeeding with their studies, decreasing their risk of mental illness, exhaustion and anxiety-related problems. Today, there is a lack of support to help students take control of their procrastination behaviours. Therefor, and due to the high prevalence of procrastination among students, it is valuable to evaluate a scalable digital procrastination support for university students.

For peer review only - http://bmjopen.bmj.com/site/about/guidelines.xhtml

Declarations

Contributors: Study objectives, outcomes, trial design and analysis plan were decided by MB and KÅ, as well as development and conceptualisation of intervention materials. KÅ drafted the protocol, which was revised by MB. Both authors contributed with intellectual content and approved the final version. KÅ and MB will be responsible for data collection and statistical analysis.

Competing interests: Author MB owns a private company (Alexit AB) that maintains and distributes evidence-based lifestyle interventions to be used by the public and in health care settings. Alexit AB played no role in developing the intervention, study design, data analysis, data interpretation, or writing of this report. Author KÅ declare no competing interests.

Funding: This trial is conducted under the auspices of the Swedish Research Council for Health, Working Life, and Welfare (Grant number 2018-01410). The funders had no role in study design, data collection and analysis, decision to publish, or preparation of the manuscript.

Data availability statement: Deidentified datasets generated during and/or analysed during the current study will be made available upon reasonable request to the corresponding author, after approval of a proposal and with a signed data access agreement.

Acknowledgements: We would like to acknowledge the student health care professionals at the participating universities who provided us with their time and shared their experiences.

For peer review only

References

1. Steel P. The nature of procrastination: a meta-analytic and theoretical review of quintessential self-regulatory failure. *Psychol Bull*. 2007;133(1):65-94. doi:10.1037/0033-2909.133.1.65, PMID:17201571
2. Fernie BA, Bharucha Z, Nikčević A V, Marino C, Spada MM. A Metacognitive model of procrastination. *J Affect Disord*. 2017;210:196-203. doi:10.1016/j.jad.2016.12.042, PMID:28056361
3. Johansson F, Rozental A, Edlund K, Côté P, Sundberg T, Onell C, Rudman A, Skillgate E. Associations Between Procrastination and Subsequent Health Outcomes Among University Students in Sweden. *JAMA Netw open*. 2023;6(1):e2249346. doi:10.1001/jamanetworkopen.2022.49346, PMID:36598789
4. Beutel ME, Klein EM, Aufenanger S, Brähler E, Dreier M, Müller KW, Quiring O, Reinecke L, Schmutzer G, Stark B, Wölfling K. Procrastination, Distress and Life Satisfaction across the Age Range - A German Representative Community Study. *PLoS One*. 2016;11(2):e0148054. doi:10.1371/journal.pone.0148054, PMID:26871572
5. Garzón-Umerenkova A, de la Fuente J, Amate J, Paoloni P V, Fadda S, Pérez JF. A Linear Empirical Model of Self-Regulation on Flourishing, Health, Procrastination, and Achievement, Among University Students. *Front Psychol*. 2018;9:536. doi:10.3389/fpsyg.2018.00536, PMID:29706922
6. Basirimoghadam M, Rafii F, Ebadi A. Self-rated health and general procrastination in nurses: a cross-sectional study. *Pan Afr Med J*. 2020;36:254. doi:10.11604/pamj.2020.36.254.23720, PMID:33014250
7. Zhang S, Liu P, Feng T. To do it now or later: The cognitive mechanisms and neural substrates underlying procrastination. *Wiley Interdiscip Rev Cogn Sci*. 2019;10(4):e1492. doi:10.1002/wcs.1492, PMID:30638308
8. Zacks S, Hen M. Academic interventions for academic procrastination: A review of the literature. *J Prev Interv Community*. 2018;46(2):117-130. doi:10.1080/10852352.2016.1198154, PMID:29485384
9. Kroese FM, de Ridder DTD. Health behaviour procrastination: a novel reasoned route towards self-regulatory failure. *Health Psychol Rev*. 2016;10(3):313-325. doi:10.1080/17437199.2015.1116019, PMID:26530737
10. Kroese FM, Evers C, Adriaanse MA, de Ridder DTD. Bedtime procrastination: A self-regulation perspective on sleep insufficiency in the general population. *J Health Psychol*. 2016;21(5):853-862. doi:10.1177/1359105314540014, PMID:24997168
11. Kelly SM, Walton HR. "I'll work out tomorrow": The Procrastination in Exercise Scale. *J Health Psychol*. 2021;26(13):2613-2625. doi:10.1177/1359105320916541, PMID:32459106
12. Atalayin C, Balkis M, Tezel H, Kayrak G. Procrastination and predictor variables among a group of dental students in Turkey. *Psychol Health Med*. 2018;23(6):726-732. doi:10.1080/13548506.2017.1418014, PMID:29262718
13. Ferrari JR, Ozer BU, Demir A. Chronic procrastination among Turkish adults: exploring decisional, avoidant, and arousal styles. *J Soc Psychol*. 2009;149(3):402-408. doi:10.3200/SOCP.149.3.402-408, PMID:19537606
14. Rozental A, Forsell E, Svensson A, Andersson G, Carlbring P. Internet-based cognitive-behavior therapy for procrastination: A randomized controlled trial. *J Consult Clin Psychol*. 2015;83(4):808-824. doi:10.1037/ccp0000023, PMID:25939016
15. Rozental A, Forsström D, Lindner P, Nilsson S, Mårtensson L, Rizzo A, Andersson G, Carlbring P. Treating Procrastination Using Cognitive Behavior Therapy: A Pragmatic Randomized Controlled Trial Comparing Treatment Delivered via the Internet or in Groups. *Behav Ther*. 2018;49(2):180-197. doi:10.1016/j.beth.2017.08.002, PMID:29530258
16. Eckert M, Ebert DD, Lehr D, Sieland B, Berking M. Does SMS-Support Make a Difference? Effectiveness of a Two-Week Online-Training to Overcome Procrastination. A Randomized

For peer review only - http://bmjopen.bmj.com/site/about/guidelines.xhtml

Controlled Trial. *Front Psychol*. 2018;9:1103. doi:10.3389/fpsyg.2018.01103, PMID:30026713
17. Gagnon J, Dionne F, Raymond G, Grégoire S. Pilot study of a Web-based acceptance and commitment therapy intervention for university students to reduce academic procrastination. *J Am Coll Health*. 2019;67(4):374-382. doi:10.1080/07448481.2018.1484361, PMID:29979942
18. Lukas CA, Berking M. Reducing procrastination using a smartphone-based treatment program: A randomized controlled pilot study. *Internet Interv*. 2018;12:83-90. doi:10.1016/j.invent.2017.07.002, PMID:30135772
19. Küchler A-M, Albus P, Ebert DD, Baumeister H. Effectiveness of an internet-based intervention for procrastination in college students (StudiCare Procrastination): Study protocol of a randomized controlled trial. *Internet Interv*. 2019;17:100245. doi:10.1016/j.invent.2019.100245, PMID:31080750
20. Chan A-W, Tetzlaff JM, Altman DG, Laupacis A, Gøtzsche PC, Krleža-Jerić K, Hróbjartsson A, Mann H, Dickersin K, Berlin JA, Doré CJ, Parulekar WR, Summerskill WSM, Groves T, Schulz KF, Sox HC, Rockhold FW, Rennie D, Moher D. SPIRIT 2013 statement: defining standard protocol items for clinical trials. *Ann Intern Med*. 2013;158(3):200-207. doi:10.7326/0003-4819-158-3-201302050-00583, PMID:23295957
21. Bendtsen P, McCambridge J, Bendtsen M, Karlsson N, Nilsen P. Effectiveness of a proactive mail-based alcohol Internet intervention for university students: dismantling the assessment and feedback components in a randomized controlled trial. *J Med Internet Res*. 2012;14(5):e142. doi:10.2196/jmir.2062, PMID:23113955
22. Michie, S.; Atkins, L.; West R. *The Behaviour Change Wheel: A Guide to Designing Interventions*. UK: Silverback publishing; 2015. http://www.behaviourchangewheel.com/.
23. Michie S, van Stralen MM, West R. The behaviour change wheel: a new method for characterising and designing behaviour change interventions. *Implement Sci*. 2011;6:42. doi:10.1186/1748-5908-6-42, PMID:21513547
24. Svartdal F, Steel P. Irrational Delay Revisited: Examining Five Procrastination Scales in a Global Sample. *Front Psychol*. 2017;8:1927. doi:10.3389/fpsyg.2017.01927, PMID:29163302
25. Kanfer FH, Gaelick-Buys L. Self-management methods. In: *Helping People Change: A Textbook of Methods, 4th Ed.* Pergamon general psychology series, Vol. 52. Elmsford, NY, US: Pergamon Press; 1991:305-360.
26. Steel P. Arousal, avoidant and decisional procrastinators: Do they exist? *Pers Individ Dif*. 2010;48(8):926-934. doi:https://doi.org/10.1016/j.paid.2010.02.025
27. Rozental A, Forsell E, Svensson A, Forsström D, Andersson G, Carlbring P. Psychometric evaluation of the Swedish version of the pure procrastination scale, the irrational procrastination scale, and the susceptibility to temptation scale in a clinical population. *BMC Psychol*. 2014;2(1):54. doi:10.1186/s40359-014-0054-z, PMID:25566392
28. Spitzer RL, Kroenke K, Williams JBW, Löwe B. A brief measure for assessing generalized anxiety disorder: the GAD-7. *Arch Intern Med*. 2006;166(10):1092-1097. doi:10.1001/archinte.166.10.1092, PMID:16717171
29. Cohen S, Kamarck T, Mermelstein R. A Global Measure of Perceived Stress. *J Health Soc Behav*. 1983;24(4):385-396. http://www.jstor.org/stable/2136404. Accessed June 13, 2022.
30. Vallejo MA, Vallejo-Slocker L, Fernández-Abascal EG, Mañanes G. Determining factors for stress perception assessed with the Perceived Stress Scale (PSS-4) in Spanish and other European samples. *Front Psychol*. 2018;9(JAN). doi:10.3389/fpsyg.2018.00037
31. Shorter GW, Bray JW, Heather N, Berman AH, Giles EL, Clarke M, Barbosa C, O'Donnell AJ, Holloway A, Riper H, Daeppen J-B, Monteiro MG, Saitz R, McNeely J, McKnight-Eily L, Cowell A, Toner P, Newbury-Birch D. The "Outcome Reporting in Brief Intervention Trials: Alcohol" (ORBITAL) Core Outcome Set: International Consensus on Outcomes to Measure in Efficacy and Effectiveness Trials of Alcohol Brief Interventions. *J Stud Alcohol Drugs*. 2021;82(5):638-646. doi:10.15288/jsad.2021.82.638, PMID:34546911
32. Socialstyrelsen. *Nationella Riktlinjer För Prevention Och Behandling Vid Ohälsosamma Levnadsvanor.*; 2018. https://www.socialstyrelsen.se/globalassets/sharepoint-

For peer review only - http://bmjopen.bmj.com/site/about/guidelines.xhtml

dokument/artikelkatalog/nationella-riktlinjer/2018-6-24.pdf.
33. Bendtsen M. A Gentle Introduction to the Comparison Between Null Hypothesis Testing and Bayesian Analysis: Reanalysis of Two Randomized Controlled Trials. *J Med Internet Res*. 2018;20(10):e10873. doi:10.2196/10873
34. Åsberg K, Eldh AC, Löf M, Bendtsen M. A balancing act-finding one´s way to health and well-being: A qualitative analysis of interviews with Swedish university students on lifestyle and behavior change. *PLoS One*. 2022;17(10):e0275848. doi:10.1371/journal.pone.0275848, PMID:36227904
35. Ebert JF, Huibers L, Christensen B, Christensen MB. Paper- or Web-Based Questionnaire Invitations as a Method for Data Collection: Cross-Sectional Comparative Study of Differences in Response Rate, Completeness of Data, and Financial Cost. *J Med Internet Res*. 2018;20(1):e24. doi:10.2196/jmir.8353, PMID:29362206
36. McCambridge J, Bendtsen M, Karlsson N, White IR, Nilsen P, Bendtsen P. Alcohol assessment and feedback by email for university students: main findings from a randomised controlled trial. *Br J Psychiatry*. 2013;203(5):334-340. doi:10.1192/bjp.bp.113.128660, PMID:24072758
37. Bendtsen M, McCambridge J. Causal models accounted for research participation effects when estimating effects in a behavioral intervention trial. *J Clin Epidemiol*. 2021;136:77-83. doi:10.1016/j.jclinepi.2021.03.008, PMID:33727133

For peer review only

FIGURE TITLES

Figure 1. Study design flowchart

Figure 2. Screenshots of the intervention

Figure 3. SPIRIT figure presenting participant timeline

For peer review only - http://bmjopen.bmj.com/site/about/guidelines.xhtml

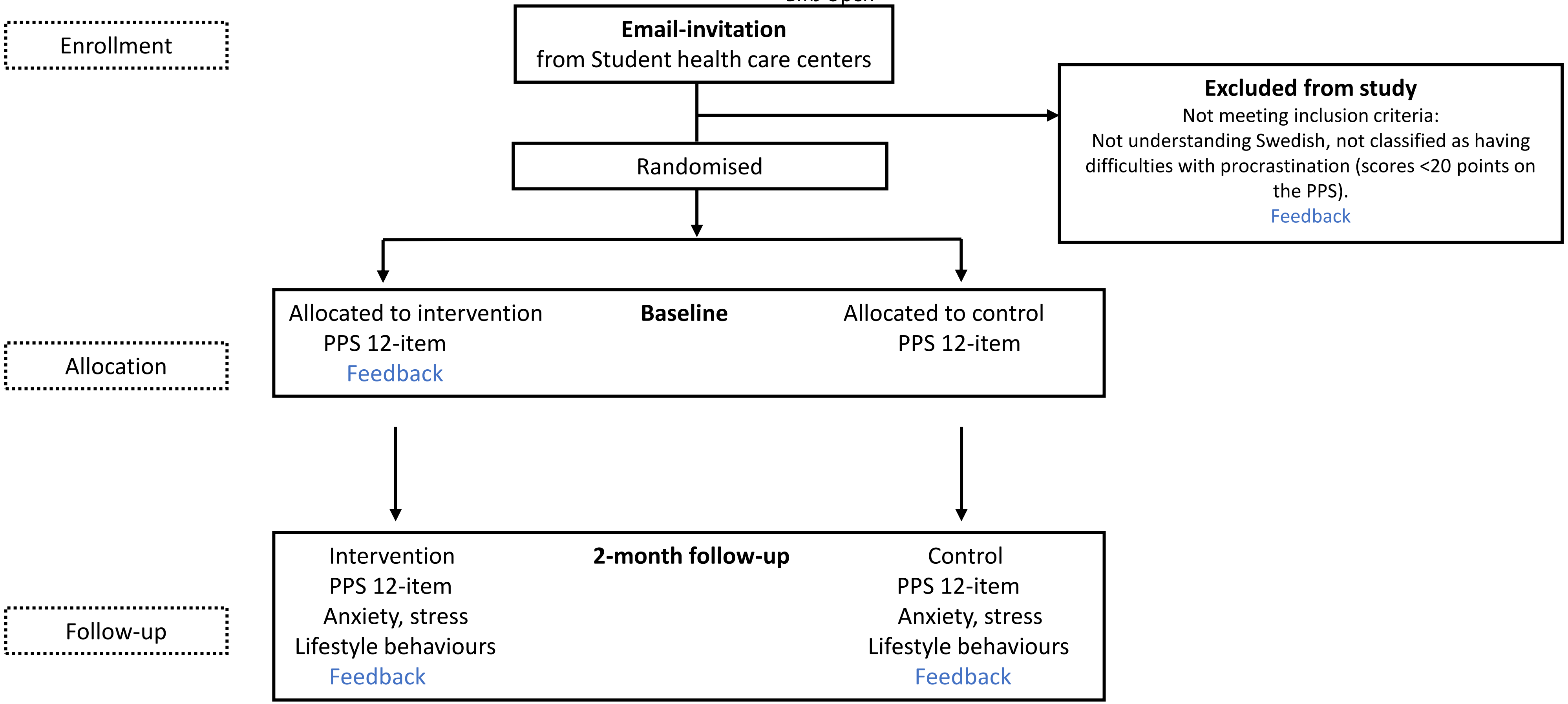
Enrollment
Email-invitation
from Student health care centers
Excluded from study
Not meeting inclusion criteria:
Not understanding Swedish, not classified as having difficulties with procrastination (scores <20 points on the PPS).
Feedback
Randomised
Allocation
Allocated to intervention
PPS 12-item
Feedback
Baseline
Allocated to control
PPS 12-item
Follow-up
Intervention
PPS 12-item
Anxiety, stress
Lifestyle behaviours
Feedback
2-month follow-up
Control
PPS 12-item
Anxiety, stress
Lifestyle behaviours
Feedback

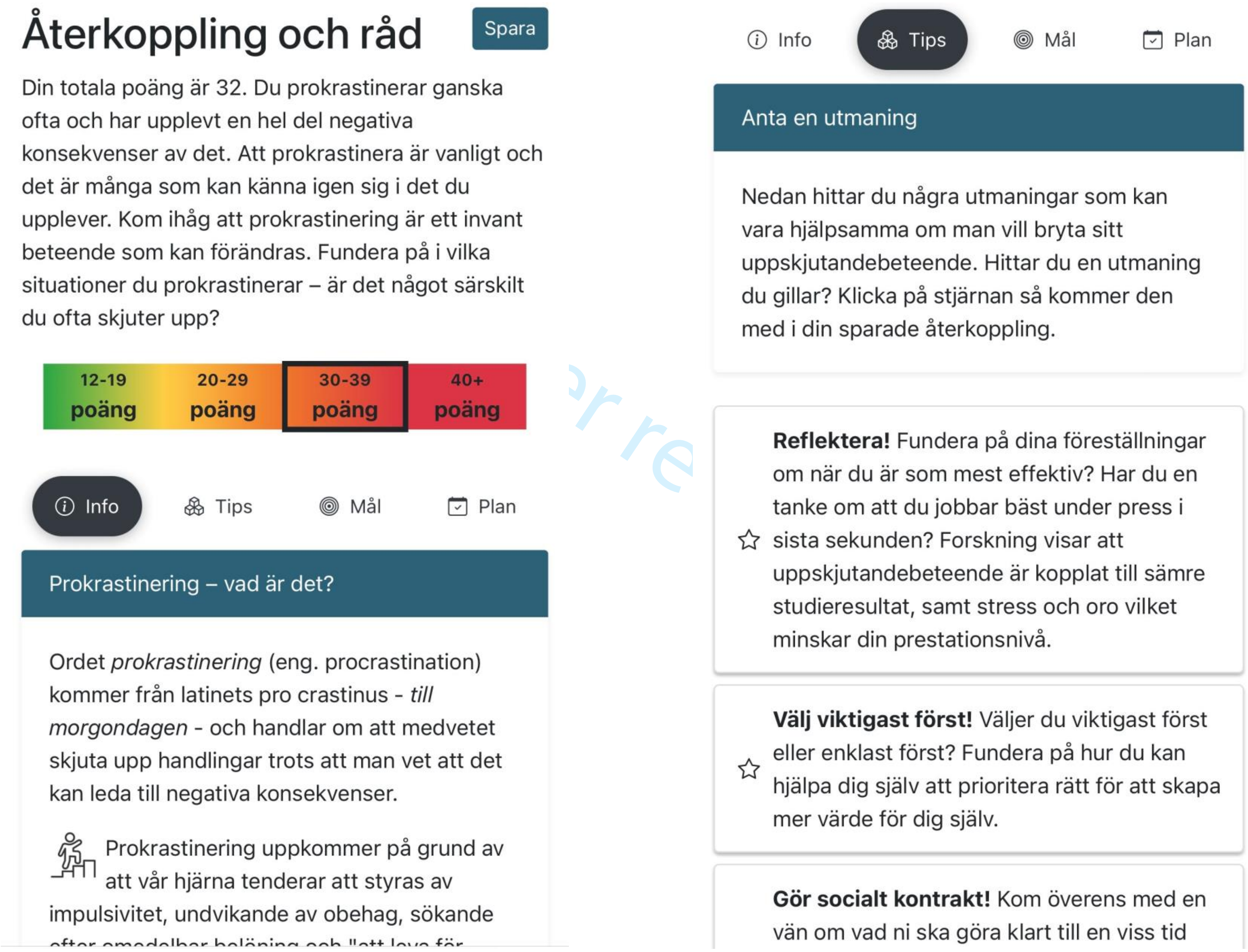
Återkoppling och råd
Spara
Din totala poäng är 32. Du prokrastinerar ganska ofta och har upplevt en hel del negativa konsekvenser av det. Att prokrastinera är vanligt och det är många som kan känna igen sig i det du upplever. Kom ihåg att prokrastinering är ett invant beteende som kan förändras. Fundera på i vilka situationer du prokrastinerar – är det något särskilt du ofta skjuter upp?
12-19 poäng
20-29 poäng
30-39 poäng
40+ poäng
Info
Tips
Mål
Plan
Prokrastinering – vad är det?
Ordet prokrastinering (eng. procrastination) kommer från latinets pro crastinus - till morgondagen - och handlar om att medvetet skjuta upp handlingar trots att man vet att det kan leda till negativa konsekvenser.
Prokrastinering uppkommer på grund av att vår hjärna tenderar att styras av impulsivitet, undvikande av obehag, sökande
Info
Tips
Mål
Plan
Anta en utmaning
Nedan hittar du några utmaningar som kan vara hjälpsamma om man vill bryta sitt uppskjutandebeteende. Hittar du en utmaning du gillar? Klicka på stjärnan så kommer den med i din sparade återkoppling.
Reflektera! Fundera på dina föreställningar om när du är som mest effektiv? Har du en tanke om att du jobbar bäst under press i sista sekunden? Forskning visar att uppskjutandebeteende är kopplat till sämre studieresultat, samt stress och oro vilket minskar din prestationsnivå.
Välj viktigast först! Väljer du viktigast först eller enklast först? Fundera på hur du kan hjälpa dig själv att prioritera rätt för att skapa mer värde för dig själv.
Gör socialt kontrakt! Kom överens med en vän om vad ni ska göra klart till en viss tid

Figure. SPIRIT figure presenting participant timeline for the Focus procrastination study

	STUDY PERIOD			
	**Enrolment**	**Allocation**	**Post-allocation**	**Close-out**
**TIMEPOINT**	**0**	**0**	**0**	***2 months***
**ENROLMENT:**				
**Informed consent**	X			
**Eligibility screen**	X			
**Allocation**		X		
**INTERVENTIONS:**				
Digital intervention		X ⟶	⟶	⟶
Control condition		X ⟶	⟶	⟶
**ASSESSMENTS:**				
Baseline questionnaire	X			
Pure procrastination scale	X			X
Lifestyle questionnaire				X
User evaluation questionnaire				X

For peer review only

APPENDIX A – INFORMED CONSENT

We would like to ask you to participate in a research project. On this page you can find information about the project and what participation entails.

WHY ARE WE DOING THIS STUDY AND WHY DO YOU WANT ME TO PARTICIPATE?

Focus is a research project which aims to estimate the effects of a brief digital procrastination intervention on procrastination, anxiety and stress symptoms, and lifestyle behaviours among university students in Sweden. The intervention is delivered online and since you are a college or university student enrolled at a university in Sweden, we would like to ask you if you would like to participate in the study.

The research project is conducted at Linköping University in Sweden, please find contact details for the primary investigator below.

WHAT IS EXPECTED OF ME?

Those who consent to take part in the study will first be asked to complete a short, online questionnaire about their current levels of procrastination. Participants will thereafter receive feedback on their current level of procrastination. Depending on which version of Focus you have been assigned to, you might also receive access to resources designed to support you change your procrastination behaviour. After two months, all participants will, again, be asked to complete a follow-up questionnaire regarding their current levels of anxiety and stress, procrastination, and lifestyle behaviours (via e-mail). The questionnaires take 5-10 minutes to complete.

WILL I BE TAKING ANY RISKS BY PARTICIPATING IN THIS STUDY?

If you decide to take part in the study then you should be aware that while Focus has been designed based on current scientific evidence regarding how to support individuals to change their procrastination behaviours, however, not everyone who uses Focus will succeed in procrastinating less. This may feel like a failure and may be de-motivating. Participants should also be aware that changing behaviours and challenging oneself to act differently, may initially result in discomfort and increased stress. These discomforts are passing, and in the long run the benefits of a more sustainable study situation will outweigh the discomforts.

You can always contact your student- or primary-healthcare centre if you feel any discomforts, want more help, or wish to consult a professional regarding your mental or physical health. You can also contact 1177 if you have any questions about your health.

WILL I BE LEAVING ANY PERSONAL INFORMATION?

The project will collect and register information about you. No unauthorized persons will have access to your answers.

Your responses to the questionnaires during the study period will be stored in a database at Linköping University. We will use an encrypted version of your e-mail address in order to connect your responses from the different questionnaires. The secret key used to decrypt your e-mail address will only be accessible by the primary investigator Marcus Bendtsen (see contact information below). Neither during analysis nor publication of findings will any information be connected to you individually.

For peer review only

When the project is complete, all e-mail addresses will be deleted. The information will be stored at Linköping University for 10 years. The data collection is for scientific research and is therefore motivated by public interest (GDPR EU 2016/679, Prop. 2017/18:298).

Linköping University is responsible for your data. According to EU:s data protection regulation you have the right to, free of charge, be given access to the data collected about you in the project, and have errors corrected. You may also request that your data be deleted or use of your data be restricted. However, the right to erasure and to limit the processing of personal data does not apply when the data is necessary for the current research. If you wish to be given access to your data, you should contact the primary investigator Marcus Bendtsen (see contact information below). The data protection officer can be reached at dataskyddsombud@liu.se. If you are dissatisfied with the way your personal data has been handled, you can file a complaint at the Swedish Authority for Privacy Protection.

HOW CAN I GET MORE INFORMATION ABOUT THE RESULTS FROM THIS STUDY?

After the study has been completed, findings will be published in scientific peer-reviewed journals. Findings at the individual level will not be traceable from these publications. We will not contact you after study completion, but you are welcome to contact us if you want information on findings or copies of published reports.

INSURANCE

As a participant of a research project at Linköping University you are included in the insurance the university has at Kammarkollegiet.

PARTICIPATION IS FREE

Participation is free and you can at any time decide to end it. If you decide to end your participation you will not be asked why, and it will not affect your studies. If you wish to end your participation you should contact the primary investigator Marcus Bendtsen (see contact information below).

PRIMARY INVESTIGATOR

Marcus Bendtsen, PhD
Docent in Medical Informatics
Senior Lecturer in Experimental Social Medicine and Public Health
Department of Health Medicine and Caring Sciences, Linköping University
marcus.bendtsen@liu.se, 013-286975

For peer review only

APPENDIX B – QUESTIONNAIRES

BASELINE

1. Sex:
    a. Female
    b. Male
    c. Nonbinary
    d. Other

2. Age (numerical measure)

PROCRASTINATION

3. I delay making decisions until it´s too late
    1) Very seldom or not true of me
    2) Rarely true of me
    3) Sometimes true of me
    4) Often true of me
    5) Very often true or true of me

4. Even after I make a decision I delay acting upon it
    1) Very seldom or not true of me
    2) Rarely true of me
    3) Sometimes true of me
    4) Often true of me
    5) Very often true or true of me

5. I waste a lot of time on trivial matters before getting to the final decisions
    1) Very seldom or not true of me
    2) Rarely true of me
    3) Sometimes true of me
    4) Often true of me
    5) Very often true or true of me

6. In preparation for some deadlines, I often waste time by doing other things
    1) Very seldom or not true of me
    2) Rarely true of me
    3) Sometimes true of me
    4) Often true of me
    5) Very often true or true of me

7. Even jobs that require little else except sitting down and doing them, I find that they seldom get done for days
    1) Very seldom or not true of me
    2) Rarely true of me
    3) Sometimes true of me
    4) Often true of me
    5) Very often true or true of me

For peer review only

8. I often find myself performing tasks that I had intended to do days before
   1) Very seldom or not true of me
   2) Rarely true of me
   3) Sometimes true of me
   4) Often true of me
   5) Very often true or true of me

9. I am continually saying ”I´ll do it tomorrow”
   1) Very seldom or not true of me
   2) Rarely true of me
   3) Sometimes true of me
   4) Often true of me
   5) Very often true or true of me

10. I generally delay before starting on work I have to do
   1) Very seldom or not true of me
   2) Rarely true of me
   3) Sometimes true of me
   4) Often true of me
   5) Very often true or true of me

11. I find myself running out of time
   1) Very seldom or not true of me
   2) Rarely true of me
   3) Sometimes true of me
   4) Often true of me
   5) Very often true or true of me

12. I don´t get things done on time
   1) Very seldom or not true of me
   2) Rarely true of me
   3) Sometimes true of me
   4) Often true of me
   5) Very often true or true of me

13. I am not very good at meeting deadlines
   1) Very seldom or not true of me
   2) Rarely true of me
   3) Sometimes true of me
   4) Often true of me
   5) Very often true or true of me

14. Putting things off till the last minute has cost me money in the past
   1) Very seldom or not true of me
   2) Rarely true of me
   3) Sometimes true of me
   4) Often true of me
   5) Very often true or true of me

For peer review only

2-MONTH FOLLOW-UP

PROCRASTINATION

1. I delay making decisions until it´s too late
   1) Very seldom or not true of me
   2) Rarely true of me
   3) Sometimes true of me
   4) Often true of me
   5) Very often true or true of me

2. Even after I make a decision I delay acting upon it
   1) Very seldom or not true of me
   2) Rarely true of me
   3) Sometimes true of me
   4) Often true of me
   5) Very often true or true of me

3. I waste a lot of time on trivial matters before getting to the final decisions
   1) Very seldom or not true of me
   2) Rarely true of me
   3) Sometimes true of me
   4) Often true of me
   5) Very often true or true of me

4. In preparation for some deadlines, I often waste time by doing other things
   1) Very seldom or not true of me
   2) Rarely true of me
   3) Sometimes true of me
   4) Often true of me
   5) Very often true or true of me

5. Even jobs that require little else except sitting down and doing them, I find that they seldom get done for days
   1) Very seldom or not true of me
   2) Rarely true of me
   3) Sometimes true of me
   4) Often true of me
   5) Very often true or true of me

6. I often find myself performing tasks that I had intended to do days before
   1) Very seldom or not true of me
   2) Rarely true of me
   3) Sometimes true of me
   4) Often true of me
   5) Very often true or true of me

7. I am continually saying ”I´ll do it tomorrow”
   1) Very seldom or not true of me
   2) Rarely true of me
   3) Sometimes true of me
   4) Often true of me
   5) Very often true or true of me

For peer review only

8. I generally delay before starting on work I have to do
    1) Very seldom or not true of me
    2) Rarely true of me
    3) Sometimes true of me
    4) Often true of me
    5) Very often true or true of me

9. I find myself running out of time
    1) Very seldom or not true of me
    2) Rarely true of me
    3) Sometimes true of me
    4) Often true of me
    5) Very often true or true of me

10. I don´t get things done on time
    1) Very seldom or not true of me
    2) Rarely true of me
    3) Sometimes true of me
    4) Often true of me
    5) Very often true or true of me

11. I am not very good at meeting deadlines
    1) Very seldom or not true of me
    2) Rarely true of me
    3) Sometimes true of me
    4) Often true of me
    5) Very often true or true of me

12. Putting things off till the last minute has cost me money in the past
    1) Very seldom or not true of me
    2) Rarely true of me
    3) Sometimes true of me
    4) Often true of me
    5) Very often true or true of me

ANXIETY AND STRESS

13. Over the last 2 weeks, how often have you been bothered by the following problems?
    1) Feeling nervous, anxious or on the edge
    2) Not being able to stop or control worrying
    3) Worrying too much about different things
    4) Trouble relaxing
    5) Being so restless that it is hard to sit still
    6) Becoming easily annoyed or irritable
    7) Feeling afraid as if something awful might happen
        a. Not at all
        b. Several days
        c. More than half of the days
        d. Nearly every day

For peer review only

14. In the last month, how often have you felt that you were unable to control the important things in your life?
    a. Never
    b. Almost never
    c. Sometimes
    d. Fairly often
    e. Very often

15. In the last month, how often have you felt confident about your ability to handle your personal problems?
    a. Never
    b. Almost never
    c. Sometimes
    d. Fairly often
    e. Very often

16. In the last month, how often have you felt that things were going your way?
    a. Never
    b. Almost never
    c. Sometimes
    d. Fairly often
    e. Very often

17. In the last month, how often have you felt difficulties were piling up so high that you could not overcome them?
    a. Never
    b. Almost never
    c. Sometimes
    d. Fairly often
    e. Very often

LIFESTYLE BEHAVIOURS

Note: Participants are reminded of the definition of a standard unit of alcohol by graphical means, as well as given visual cues for what constitutes a unit of sugary drinks.

18. How many standard drinks of alcohol did you consume last week? (numerical measure)

19. How often, during the past month, have you consumed four or more standard drinks of alcohol on one occasion? (numerical measure)

20. How many cans (33 cl, one standard can) of sugary drinks (e.g. soft drinks, “energy drinks”) did you consume last week?

    a. 0 cans
    b. 1 can per week
    c. 2-3 cans per week
    d. 4-6 cans per week
    e. 1 can per day
    f. 1.5 cans per day
    g. 2.0 cans per day
    h. 2.5 cans per day
    i. 3.0 cans per day or more

For peer review only

21. How many portions of candy, chocolate, pastry (e.g. buns, muffins, cookies), ice cream and salty snacks (e.g. crisps, nuts, chees doodles) did you eat last week? *One portion is 50 g candy (9 pieces), 40 g chocolate (6 pieces/squares), 1 bun, 2 dl (scoops) of ice cream or 2 dl snacks (40 g)*.

a. 0 portions
b. 1 portion per week
c. 2-3 portions per week
d. 4-6 portions per week
e. 1 portion per day
f. 1.5 portions per day
g. 2.0 portions per day
h. 2.5 portions per day
i. 3.0 portions per day
j. 3.5 portions per day
k. 4.0 portions per day or more

22. How much time in total did you spend on moderate physical activity (e.g. bicycling or walking for transport or leisure) last week?

a. 0
b. Less than 30 minutes
c. 30-60 minutes
d. 1 hours
e. 1.5 hours
f. 2 hours
g. 2.5 hours
h. 3 hours
i. 3.5 hours (i.e. 30 minutes per day)
j. 4 hours
k. 5 hours
l. 6 hours
m. 7 hours (i.e. 1 hour per day)
n. 10.5 hours (i.e. 1.5 hours per day)
o. 14 hours (i.e. 2 hours per day)

23. How much time in total did you spend on vigorous physical activity (i.e. producing fast increases in breathing or heart rate), for instance running, aerobics, etc. last week?

a. 0
b. Less than 30 minutes
c. 30-60 minutes
d. 1 hours
e. 1.5 hours
f. 2 hours
g. 2.5 hours
h. 3 hours
i. 3.5 hours (i.e. 30 minutes per day)
j. 4 hours
k. 5 hours
l. 6 hours
m. 7 hours (i.e. 1 hour per day)
n. 10.5 hours (i.e. 1.5 hours per day)
o. 14 hours (i.e. 2 hours per day)

For peer review only

PERCEIVED USEFULNESS AND GENERAL OPINION OF THE SUPPORT RECEIVED

24. Overall, how well did the support given to you suit your needs?
(1 = "Not very helpful" to 5 = "Very helpful")

25. Please leave a comment describing your needs and how the support did or did not address them (Free-text).

26. Do you believe that the support given to you would be helpful for other individuals that want to change their procrastination behaviour?
(1 = "Not very helpful" to 5 = "Very helpful")

For peer review only

SPIRIT
STANDARD PROTOCOL ITEMS: RECOMMENDATIONS FOR INTERVENTIONAL TRIALS

SPIRIT 2013 Checklist: Recommended items to address in a clinical trial protocol and related documents*

Section/item	Item No	Description	Addressed on page number
**Administrative information**			
Title	1	Descriptive title identifying the study design, population, interventions, and, if applicable, trial acronym	______1______
Trial registration	2a	Trial identifier and registry name. If not yet registered, name of intended registry	_____1,2______
	2b	All items from the World Health Organization Trial Registration Data Set	_____1,2______
Protocol version	3	Date and version identifier	______NA____
Funding	4	Sources and types of financial, material, and other support	_____ 12_____
Roles and responsibilities	5a	Names, affiliations, and roles of protocol contributors	_____ 1______
	5b	Name and contact information for the trial sponsor	_____12____
	5c	Role of study sponsor and funders, if any, in study design; collection, management, analysis, and interpretation of data; writing of the report; and the decision to submit the report for publication, including whether they will have ultimate authority over any of these activities	_____12____
	5d	Composition, roles, and responsibilities of the coordinating centre, steering committee, endpoint adjudication committee, data management team, and other individuals or groups overseeing the trial, if applicable (see Item 21a for data monitoring committee)	_____NA_____

For peer review only

**Introduction**			
Background and rationale	6a	Description of research question and justification for undertaking the trial, including summary of relevant studies (published and unpublished) examining benefits and harms for each intervention	_____3______
	6b	Explanation for choice of comparators	_____3, 4, 5_____
Objectives	7	Specific objectives or hypotheses	_____4_______
Trial design	8	Description of trial design including type of trial (eg, parallel group, crossover, factorial, single group), allocation ratio, and framework (eg, superiority, equivalence, noninferiority, exploratory)	_____4______
**Methods: Participants, interventions, and outcomes**			
Study setting	9	Description of study settings (eg, community clinic, academic hospital) and list of countries where data will be collected. Reference to where list of study sites can be obtained	_____4_______
Eligibility criteria	10	Inclusion and exclusion criteria for participants. If applicable, eligibility criteria for study centres and individuals who will perform the interventions (eg, surgeons, psychotherapists)	_____4______
Interventions	11a	Interventions for each group with sufficient detail to allow replication, including how and when they will be administered	____4,5_______
	11b	Criteria for discontinuing or modifying allocated interventions for a given trial participant (eg, drug dose change in response to harms, participant request, or improving/worsening disease)	_____NA______
	11c	Strategies to improve adherence to intervention protocols, and any procedures for monitoring adherence (eg, drug tablet return, laboratory tests)	_____NA_____
	11d	Relevant concomitant care and interventions that are permitted or prohibited during the trial	_____NA______
Outcomes	12	Primary, secondary, and other outcomes, including the specific measurement variable (eg, systolic blood pressure), analysis metric (eg, change from baseline, final value, time to event), method of aggregation (eg, median, proportion), and time point for each outcome. Explanation of the clinical relevance of chosen efficacy and harm outcomes is strongly recommended	_____6,7______
Participant timeline	13	Time schedule of enrolment, interventions (including any run-ins and washouts), assessments, and visits for participants. A schematic diagram is highly recommended (see Figure)	__7, Figure 3__

For peer review only - http://bmjopen.bmj.com/site/about/guidelines.xhtml

Sample size	14	Estimated number of participants needed to achieve study objectives and how it was determined, including clinical and statistical assumptions supporting any sample size calculations	_____7______
Recruitment	15	Strategies for achieving adequate participant enrolment to reach target sample size	_____7,8______
**Methods: Assignment of interventions (for controlled trials)**			
Allocation:			
Sequence generation	16a	Method of generating the allocation sequence (eg, computer-generated random numbers), and list of any factors for stratification. To reduce predictability of a random sequence, details of any planned restriction (eg, blocking) should be provided in a separate document that is unavailable to those who enrol participants or assign interventions	_______8_______
Allocation concealment mechanism	16b	Mechanism of implementing the allocation sequence (eg, central telephone; sequentially numbered, opaque, sealed envelopes), describing any steps to conceal the sequence until interventions are assigned	_____8________
Implementation	16c	Who will generate the allocation sequence, who will enrol participants, and who will assign participants to interventions	_____8________
Blinding (masking)	17a	Who will be blinded after assignment to interventions (eg, trial participants, care providers, outcome assessors, data analysts), and how	_____8________
	17b	If blinded, circumstances under which unblinding is permissible, and procedure for revealing a participant's allocated intervention during the trial	_____8________
**Methods: Data collection, management, and analysis**			
Data collection methods	18a	Plans for assessment and collection of outcome, baseline, and other trial data, including any related processes to promote data quality (eg, duplicate measurements, training of assessors) and a description of study instruments (eg, questionnaires, laboratory tests) along with their reliability and validity, if known. Reference to where data collection forms can be found, if not in the protocol	____4,7________
	18b	Plans to promote participant retention and complete follow-up, including list of any outcome data to be collected for participants who discontinue or deviate from intervention protocols	_____7,8_______

Data management	19	Plans for data entry, coding, security, and storage, including any related processes to promote data quality (eg, double data entry; range checks for data values). Reference to where details of data management procedures can be found, if not in the protocol	___NA_________
Statistical methods	20a	Statistical methods for analysing primary and secondary outcomes. Reference to where other details of the statistical analysis plan can be found, if not in the protocol	____8,9________
	20b	Methods for any additional analyses (eg, subgroup and adjusted analyses)	____8,9_______
	20c	Definition of analysis population relating to protocol non-adherence (eg, as randomised analysis), and any statistical methods to handle missing data (eg, multiple imputation)	____8,9_______
**Methods: Monitoring**			
Data monitoring	21a	Composition of data monitoring committee (DMC); summary of its role and reporting structure; statement of whether it is independent from the sponsor and competing interests; and reference to where further details about its charter can be found, if not in the protocol. Alternatively, an explanation of why a DMC is not needed	_____NA______
	21b	Description of any interim analyses and stopping guidelines, including who will have access to these interim results and make the final decision to terminate the trial	_____NA______
Harms	22	Plans for collecting, assessing, reporting, and managing solicited and spontaneously reported adverse events and other unintended effects of trial interventions or trial conduct	_____NA_______
Auditing	23	Frequency and procedures for auditing trial conduct, if any, and whether the process will be independent from investigators and the sponsor	_____NA_______
**Ethics and dissemination**			
Research ethics approval	24	Plans for seeking research ethics committee/institutional review board (REC/IRB) approval	______2,9_____
Protocol amendments	25	Plans for communicating important protocol modifications (eg, changes to eligibility criteria, outcomes, analyses) to relevant parties (eg, investigators, REC/IRBs, trial participants, trial registries, journals, regulators)	______NA_____

For peer review only - http://bmjopen.bmj.com/site/about/guidelines.xhtml

Consent or assent	26a	Who will obtain informed consent or assent from potential trial participants or authorised surrogates, and how (see Item 32)	______4, App A___
	26b	Additional consent provisions for collection and use of participant data and biological specimens in ancillary studies, if applicable	______N/A______
Confidentiality	27	How personal information about potential and enrolled participants will be collected, shared, and maintained in order to protect confidentiality before, during, and after the trial	______App A______
Declaration of interests	28	Financial and other competing interests for principal investigators for the overall trial and each study site	______12______
Access to data	29	Statement of who will have access to the final trial dataset, and disclosure of contractual agreements that limit such access for investigators	______12________
Ancillary and post-trial care	30	Provisions, if any, for ancillary and post-trial care, and for compensation to those who suffer harm from trial participation	______NA_______
Dissemination policy	31a	Plans for investigators and sponsor to communicate trial results to participants, healthcare professionals, the public, and other relevant groups (eg, via publication, reporting in results databases, or other data sharing arrangements), including any publication restrictions	______ 9, App A__
	31b	Authorship eligibility guidelines and any intended use of professional writers	______12_______
	31c	Plans, if any, for granting public access to the full protocol, participant-level dataset, and statistical code	______ 12 ______
**Appendices**			
Informed consent materials	32	Model consent form and other related documentation given to participants and authorised surrogates	______App A___
Biological specimens	33	Plans for collection, laboratory evaluation, and storage of biological specimens for genetic or molecular analysis in the current trial and for future use in ancillary studies, if applicable	______NA_______

*It is strongly recommended that this checklist be read in conjunction with the SPIRIT 2013 Explanation & Elaboration for important clarification on the items. Amendments to the protocol should be tracked and dated. The SPIRIT checklist is copyrighted by the SPIRIT Group under the Creative Commons "Attribution-NonCommercial-NoDerivs 3.0 Unported" license.
